# Chemoablated mouse seminiferous tubular cells enriched for very small embryonic-like stem cells undergo spontaneous spermatogenesis *in vitro*

**DOI:** 10.1186/s12958-015-0031-2

**Published:** 2015-04-18

**Authors:** Sandhya Anand, Hiren Patel, Deepa Bhartiya

**Affiliations:** Stem Cell Biology Department, National Institute for Research in Reproductive Health, Mumbai, 400 012 India

**Keywords:** Testis, VSELs, SSCs, Sperm, ES cells, PGCs

## Abstract

**Background:**

Extensive research is ongoing to empower cancer survivors to have biological parenthood. For this, sperm are cryopreserved prior to therapy and in younger children testicular biopsies are cryopreserved with a hope to mature the germ cells into sperm later on for assisted reproduction. In addition, lot of hope was bestowed on pluripotent embryonic and induced pluripotent stem cells to differentiate into sperm and oocytes. However, obtaining functional gametes from pluripotent stem cells still remains a distant dream and major bottle-neck appears to be their inefficient differentiation into primordial germ cells (PGCs). There exists yet another population of pluripotent stem cells termed very small embryonic-like stem cells (VSELs) in adult body organs including gonads. We have earlier reported that busulphan (25 mg/Kg) treatment to 4 weeks old mice destroys actively dividing cells and sperm but VSELs survive and differentiate into sperm when a healthy niche is provided *in vivo*.

**Methods:**

Mouse testicular VSELs that survived busulphan treatment were cultured for 3 weeks. A mix of surviving cells in seminiferous tubules (VSELs, possibly few spermatogonial stem cells and Sertoli cells) were cultured using Sertoli cells conditioned medium containing fetal bovine serum, follicle stimulating hormone and with no additional growth factors.

**Results:**

Stem cells underwent proliferation and clonal expansion in culture and spontaneously differentiated into sperm whereas Sertoli cells attached and provided a somatic support. Transcripts specific for various stages of spermatogenesis were up-regulated by qRT-PCR studies on day 7 suggesting VSELs (*Sca1*) and SSCs (*Gfra*) proliferate (*Pcna*), undergo spermatogenesis (spermatocyte specific marker *prohibitin*), meiosis (*Scp3*) and differentiate into sperm (post-meiotic marker *protamine*).

**Conclusions:**

Process of spermatogenesis and spermiogenesis was replicated *in vitro* starting with testicular cells that survived busulphan treatment. We have earlier reported similar ability of ovarian VSELs enriched in the ovary surface epithelial cells to form oocyte-like structures *in vitro*. This striking potential of spontaneous differentiation of primitive testicular cells including VSELs that survive chemotherapy is being described for the first time in the present study.

**Electronic supplementary material:**

The online version of this article (doi:10.1186/s12958-015-0031-2) contains supplementary material, which is available to authorized users.

## Background

Pluripotent stem cells (PSCs) including embryonic stem (ES) and induced pluripotent stem (iPS) cells have been studied for differentiation into gametes but the goal to achieve normal gametes to treat infertility still remains a distant dream [1]. The progress made in the field over the last decade in this area was recently reviewed by several groups [2-5]. PSCs easily differentiate into germ cells but their further differentiation into functional, haploid gametes remains highly inefficient. Mouse ES cells have been differentiated into both oocyte-like structures [6] and sperm-like structures [7] with the birth of live offspring using male germ cells after intra cytoplasmic sperm injection. However, the pups born were phenotypically abnormal and died pre-maturely, possibly because of abnormal epigenetic status of the gametes generated *in vitro*. By genetic modifications, human ES and iPS cells were differentiated into germ cells that underwent complete meiosis [8,9]. Eguizabal et al [10] achieved complete meiosis using human iPS cells, but the efficiency remains relatively low.

Against this background, there is yet another novel PSCs population which exists in various adult organs [11] including gonads [12,13]. These are termed very small embryonic-like stem cells (VSELs) and have been well-characterized in mouse and human testicular tissue [11,14,15]. They are distinctly spherical cells, very small in size (3 - 5 μm) with high nucleo-cytoplasmic ratio. They are very few in number and exist in relatively quiescent state under steady-state conditions, possibly because of partially erased imprinting and over-expression of cell cycle inhibitory genes [16]. Besides VSELs, body organs also harbor tissue-specific progenitor stem cells which are slightly bigger in size, proliferate rapidly, undergo clonal expansion and further differentiate into tissue specific cell types e.g. hematopoietic (HSCs) and mesenchymal (MSCs) stem cells in bone marrow, spermatogonial stem cells (SSCs) in testis, ovarian stem cells in ovary (OSCs) etc. Under stress conditions like exposure to neurotoxins, stroke, retinal damage, skin burns, streptozotocin treatment etc. VSELs readily enter cell cycle, expand in numbers and are mobilized in circulation [17,18]. Transcripts specific to the damaged organ can also be detected in the mobilized cells i.e. VSELs divide and also give rise to tissue-specific progenitors. It is this property of VSELs of getting mobilized in busulphan and cyclophosphamide treated mice (resulting in compromised gonadal function) that germ cell markers were possibly reported in circulation [19] leading to the conclusion that bone marrow is a source of germ cells. Ratajczak’s group has also proposed that VSELs are possibly the embryonic remnants that result in various cancers in adults [20] and we have recently discussed their involvement in adult ovarian biology, failure, menopause and cancer [21].

LIN-/CD45-/SCA1+ VSELs comprise 0.03 ± 0.002% of cells in adult mouse testis. Immuno-phenotyping studies reveal that unlike bigger spermatogonial stem cells (SSCs) which are more than 10 μm in size and GFRA positive, VSELs are smaller in size, negative for GFRA and express pluripotent transcripts including nuclear OCT-4. VSELs are possibly the pluripotent primordial germ cells which survive in adult testis in small numbers. They undergo asymmetric cell division to self-renew themselves and also give rise to SSCs which express cytoplasmic OCT-4, divide rapidly and further differentiate into sperm [15]. VSELs could be the cancer initiating cells in testis since nuclear OCT-4 is a specific and sensitive marker for testicular tumors [22,23] and also may have been responsible for the appearance of ES-like colonies when testicular tissue was cultured by various groups [24].

Of the four different doses of busulphan used by us to treat mice in an earlier study, the dose of 25 mg/Kg resulted in nearly complete testicular ablation associated with significant loss of Dazl and Gfra at both mRNA and protein level [14]. However, VSELs survived due to their relatively quiescent nature and flow cytometry studies revealed that VSELs numbers increased from 0.03 ± 0.002% in normal testis to 0.06 ± .004% after busulphan treatment. Further the surviving testicular VSELs were able to restore spermatogenesis *in vivo* on inter-tubular transplantation of healthy Sertoli or mesenchymal cells [see Additional file 1 for further details]. The increase in number of testicular VSELs in busulphan treated testis was similar to an earlier report where Ratajczak et al [25] reported that VSELs survive total body irradiation in mouse bone marrow and are increased in numbers as evident by increased BrdU uptake by flow cytometry. A significant depletion of hematopoietic stem cells (HSCs) was observed by them after radiotherapy similar to a loss of SSCs in testis observed by us after busulphan treatment. Possibly the chemo- and radiotherapy destroys the micro-environment (niche supporting the stem cells) in both the bone marrow and testis, and as a result the surviving VSELs are able to proliferate and increase in numbers but cannot differentiate (since the growth factors/cytokines required for differentiation are not available due to the compromised nature of the niche). Recently it was reported by our group that busulphan and cyclophosphamide treatment depletes mice ovaries of follicular reserve but VSELs survive, increase in numbers in response to follicle stimulating hormone treatment and also undergo spontaneous differentiation *in vitro* into oocyte-like structures [26]. Our group has also earlier reported that *in vitro* culture of VSELs enriched from adult mammalian (human, sheep, monkey and rabbit) ovary surface epithelium spontaneously differentiate into oocyte-like structures in a very simple culture medium (with no additional cocktail of growth factors) within three weeks; whereas the epithelial cells differentiate into mesenchymal-like fibroblasts and act essentially as a source of growth factors and cytokines required for the differentiation of oocyte-like structures [27,28].

The aim of the present study was to study the *in vitro* differentiation potential of surviving stem cells collected from busulphan treated mouse testis. For this, cells collected by enzymatic digestion of seminiferous tubules (VSELs, possibly few spermatogonial stem cells and Sertoli cells) were used to establish primary cultures. Results show that the whole process of spermatogenesis gets replicated *in vitro* in basic culture medium.

## Methods

The study was approved by the Institute’s Animal Ethics Committee (IAEC). Adult male Swiss mice maintained in the Institute experimental animal facility were used for the study. They were housed in a temperature and humidity controlled room on a 12 hour light/12 hour darkness cycle with free access to food and water. Eight weeks old Swiss mice were treated with busulphan (25 mg/Kg body weight through intraperitoneal route; body weight; Sigma-Aldrich, USA). One month after the treatment, mice were sacrificed by cervical dislocation; testes were collected and further processed for the study.

As reported earlier by our group, this dose of busulphan caused effective loss of SSCs and germ cell aplasia evidenced by histology, significant reduction of transcripts specific for SSCs (Gfra) and germ cells (*Dazl )* and also at protein level for DAZL. Besides the Sertoli cells, relatively quiescent VSELs were found to persist and increase in numbers as confirmed by flow cytometry and higher expression of specific transcripts *Oct-4A* and Sca-1 by qRT-PCR studies [14, Additional file 1].

### Preparation of testicular cell suspension

Testes were washed with phosphate buffer saline (PBS) supplemented with penicillin 100 U/ml and streptomycin 100 mg/ml. All reagents were from Invitrogen (USA) unless otherwise specified. Single cell suspension of testicular cells was obtained by a two-step enzymatic process as described earlier [14]. Briefly, this involved detunication of testes, washing the tubules in PBS followed by sequential enzymatic digestion with 1mg/ml collagenase IV and 0.25% trypsin EDTA and pipetting. The cell suspension was filtered through 40 μm cell strainer (BD Falcon; USA). This single cell suspension was washed twice again with PBS by centrifugation at 1000 g at 4°C for 10 minutes and cells were used to determine viability and for *in vitro* culture. Initial cell viability was always found to be greater than 95% by trypan blue exclusion method.

The cells suspension obtained by digesting the testicular tissue was heterogeneous in nature and different strategies were used to further enrich the surviving VSELs including (i) differential plating i.e. cells suspension was plated in 35 mm culture dish, Sertoli cells attached overnight and unattached floating cells were collected next day for the study (ii) immuno-magnetic enrichment of SCA-1 sorted cells (described below) (iii) differential centrifugation, which involved initial centrifugation at 500 rpm for 10 minutes that allowed larger cells to settle down and VSELs in supernatant were later pelleted down at a higher speed of 1000 g and used for *in vitro* culture.

### Magnetic sorting of SCA-1 positive cells from busulphan treated mice testes

SCA-1 positive cells were magnetically separated using SCA-1-FITC antibody (BD Biosciences, USA) and Easysep kit (Stem Cell Technology, USA) according to manufacturer’s instructions. Briefly, the mouse testicular cell suspension was sequentially incubated with blocking solution (provided in the kit), SCA-1 antibody, selection cocktail and magnetic nanoparticles. Then, the cell suspension was placed on a magnet and the positive cells were selected. Magnetically enriched SCA-1 positive cells were used for *in vitro* culture.

### Preparation of Sertoli cells conditioned medium

Sertoli cells are known to provide various growth factors in a paracrine manner for germ cells to undergo spermatogenesis [14]. Hence a medium conditioned with Sertoli cells was used for the present study. To obtain conditioned media, Sertoli cells from normal adult mice were cultured to confluence. Fresh medium (DMEM F12 with 10% FBS) was added and harvested after overnight incubation with Sertoli cells. This media was filtered and stored at -20^°^C till further use.

### In vitro culture

All cultures were carried out in 35 mm dish and maintained at 37°C in humidified 5% CO_2_ atmosphere. Cells for initial seeding, obtained and enriched for VSELs as described above, were cultured using Sertoli cells conditioned media supplemented with 10% FBS, 100 U/mL penicillin and 100 μg/mL streptomycin and follicle stimulating hormone (5 IU/ml). During culture, the media was changed partially every second day and care was taken not to disturb the cells in culture too much. The cultures were monitored over time and images were captured under inverted microscope (TE200, NIKON, Japan). The cultures were established five times.

### RNA isolation and cDNA synthesis

Testicular cells were fixed in Trizol (Invitrogen) for RNA extraction at the start of culture (D0) and on day 7 (D7) after culture. Total RNA was extracted using standard protocol and was treated with DNase I (Fermentas Life Sciences, USA) to remove any genomic DNA contamination. First-strand cDNA was synthesized using the iScript cDNA synthesis Kit (Bio-Rad, USA) according to the manufacturer’s instructions. Briefly, 1 μg of total RNA was incubated with 5X iScript reaction mix and reverse transcriptase mix. The reaction was carried out in G-STORM thermocycler (Gene Technologies, UK). The reaction mix was first incubated at 25°C for 5minutes, then at 42°C for 30 minutes and finally at 85°C for 5 minutes.

### RT PCR studies

PCR was performed to confirm the presence of spermatogenesis specific transcripts including *Dazl* (germ cell), *Gfra* (spermatogonial stem cell), *prohibitin* (meiotic) and *protamine* (post-meiotic) markers using specific primers and PCR conditions (Table 1). cDNA mix (2 μl) was amplified using 10 pmol of each primer, 1 U of Taq DNA polymerase (Fermentas), 1.5 mM MgCl2, and 0.4 mM dNTPs in a 25 ml reaction volume in a G-STORM thermocycler. Amplification was carried out for 35 cycles, with each cycle consisting of denaturation at 94°C for 30 seconds, annealing at the specified temperature for each set of primers for 30 seconds, elongation for 30 seconds and final extension at 72°C for 7 minutes. The products were analyzed on 1.5% agarose gel stained with 0.5 mg/ml ethidium bromide (Bangalore Genei; India) and visualized under ultraviolet transillumination. The product size was approximated using a 100-bp DNA ladder (Bangalore Genei). The negative control did not include cDNA in the reaction mixture.

**Table 1:** **Primer sequences for various transcripts used in the study**

**Primer**	**Sequence**	**Annealing temperature**
Sca-1		
F	AGAGGAAGTTTTATCTGTGCAGCCC	66°C
R	TCCACAATAACTGCTGCCTCCTGA	
Gfra		
F	GGCCTACTCGGGACTGATTGG	58°C
R	GGGAGGAGCAGCCATTGATTT	
Prohibitin		
F	GTGGCGTACAGGACATTGTG	58°C
R	AGCTCTCGCTGGGTAATCAA	
Protamine		
F	GGCCACCACCACCACAGACACAGGCG	66°C
R	TTAGTGATGGTGCCTCCTACATTTCC	
Dazl		
F	GTGTGTCGAAGGGCTATGGAT	61°C
R	ACAGGCAGCTGATATCCAGTG	
PCNA		
F	5′ GATGCCGTCGGGTGAATTTG 3′	55°C
R	5′ TCTCTATGGTTACCGCCTCCT 3′	
SCP3		
F	5′ TGTTGCAGCAGTGGGAACTGGAT 3′	68°C
R	5′ CCATCTCTTGCTGCTGAGTTTCCA 3′	
18s		
F	5′ GTCCCGTAGACAAAATGGTGA 3′	58°C
R	5′ TGCATTGCTGACAATCTTGAG 3′	
Gapdh		
F	GTCCCGTAGACAAAATGGTGA	58°C
R	TGCATTGCTGACAATCTTGAG	

### Quantitative RT-PCR studies

The expression levels of various gene transcripts were studied on day 7 compared to initial cells that were put in culture using specific primers (Table 1) by qRT-PCR studies. This included *Sca-1, Gfra, Pcna, Scp3, prohibitin and protamine* with 18s as housekeeping gene using CFX96 real-time PCR system (Bio-Rad Laboratories, USA) and SYBR Green chemistry (Bio-Rad). The amplification conditions included initial denaturation at 94°C for 3 minutes followed by 40 cycles comprising of denaturation at 94°C for 10 seconds, primer annealing for 20 seconds, and extension at 72°C for 30 seconds followed by melt curve analysis step from 55°C to 95°C. The fluorescence emitted at each cycle was collected during the extension step of each cycle. The homogeneity of the PCR amplicons was verified by running the products on 1.5% agarose gels and also by studying the melt curve. All PCR amplifications were carried out in duplicate. Mean Ct values generated in each experiment using the CFX Manager software (Bio-Rad) were used to calculate the mRNA expression levels. The fold change was calculated using ΔΔCt method. The Q-PCR studies were done on two independent cultures and results analyzed separately.

## Results

The culture experiments were repeated five times using cells suspension from chemoablated testis with VSELs enriched using three different approaches as described in the Methods section. We obtained similar results however initial presence of few spermatogonial cells which may also have survived busulphan treatment cannot be ruled out in our generic cultures. This was anyways not an issue of great concern to us as we have earlier reported that VSELs are the quiescent stem cells which undergo asymmetric cell division to give rise to the SSCs which further undergo symmetric cell divisions, proliferate rapidly, differentiate, undergo meiosis to produce sperm [16]. The underlying reasoning behind our belief that VSELs give rise to SSCs is the different OCT-4 staining pattern observed in the two cell types (nuclear vs cytoplasmic). Nuclear OCT-4 is a transcription factor required to maintain pluripotent state and when differentiation is initiated, OCT-4 is no longer required, shifts to cytoplasm and gets eventually degraded [13-15]. Thus in our present study we expected VSELs will always give rise to SSCs which will further differentiate, undergo meiosis and produce sperm.

**Figure 1:**
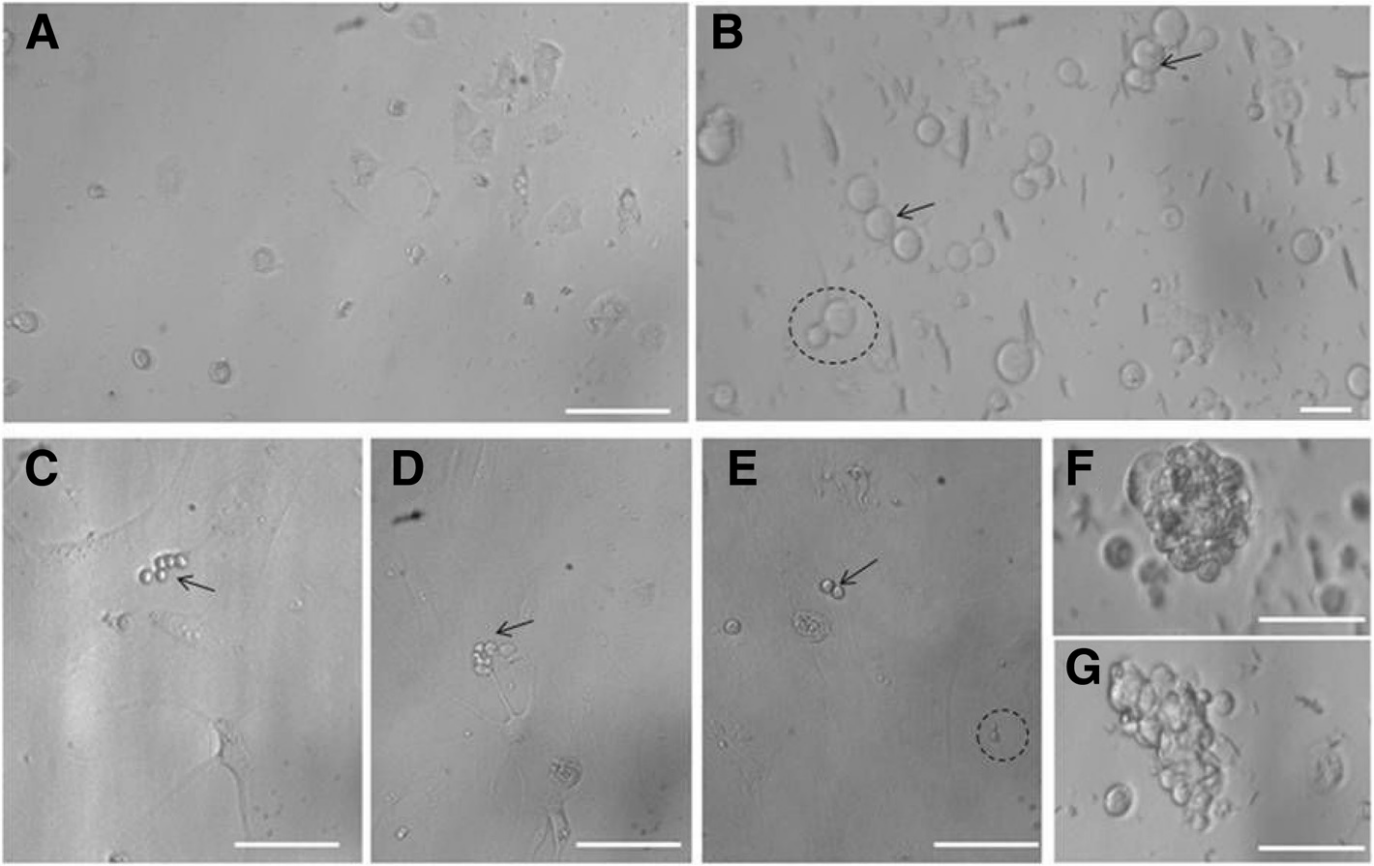
Testicular cells from chemoablated mouse after overnight culture. **(A)** Cells obtained after two-step enzymatic digestion of seminiferous tubules from busulphan treated mice were cultured. After overnight culture, two distinct cell types including attached polygonal Sertoli cells and few spherical stem cells (which remain unattached) were clearly visualized. **(B)** At higher magnification, spherical stem cells of two distinct sizes were observed. VSELs (small sized) appeared to give rise to bigger sized spermatogonial stem cells (SSCs, broken circle) and the SSCs divide rapidly and form chains (arrow). Results are in agreement with earlier proposed concept [15] that upon differentiation, small sized pluripotent VSELs with nuclear OCT-4 give rise to slightly bigger ‘progenitors’ with cytoplasmic OCT-4. VSELs undergo asymmetric cell division including self-renewal and giving rise to slightly bigger cells (broken circle) whereas the progenitors undergo rapid symmetrical divisions (arrow). **(C-G)** On day 3, the Sertoli cells appear well attached to the bottom of the culture dish as mesenchymal-like fibroblasts and apparently provide a somatic feeder support to the germ cells. Germ cells (arrow, remain unattached) were observed in close vicinity of Sertoli cells. We also observed few floating germ cell clusters which reflect clonal expansion of progenitors. Similar germ cells clusters were also reported in adult mouse ovary [13].

**Figure 2:**
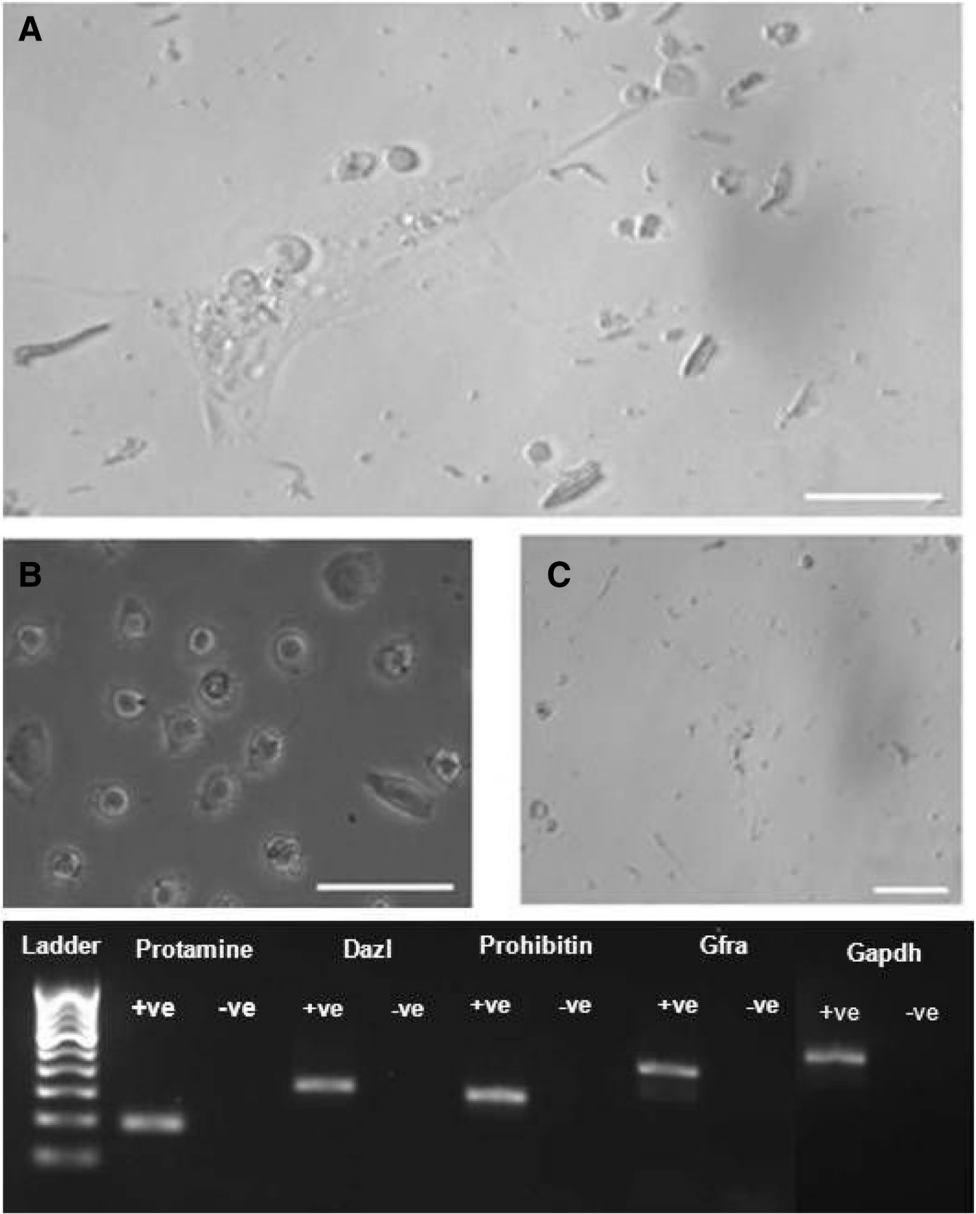
Testicular cells from chemoablated mouse in culture. **(A)** Higher magnification of cells in culture to show a close association of differentiating germ cells with the attached Sertoli cells which resemble mesenchymal fibroblasts. **(B)** Different sized cells were observed in the culture suggestive of the presence of meiotic and post-meiotic germ cells. At places, initial stages of spermiogenesis were observed including spermatids in different stages of development. These cells which appear over time as a result of differentiation were completely lacking in initial cultures. **(C)** Sperm were observed after 2 weeks of culture. Lower panel. RT-PCR analysis of RNA isolated from cells harvested on Day 7 cultures showed presence of Gfra, Dazl, protamine, prohibitin suggestive of presence of spermatogonial stem cells, germ cells, spermatocytes and sperm in culture. These cultures were neither homogenous nor synchronized, rather comprised cells in various stages of differentiation.

**Figure 3:**
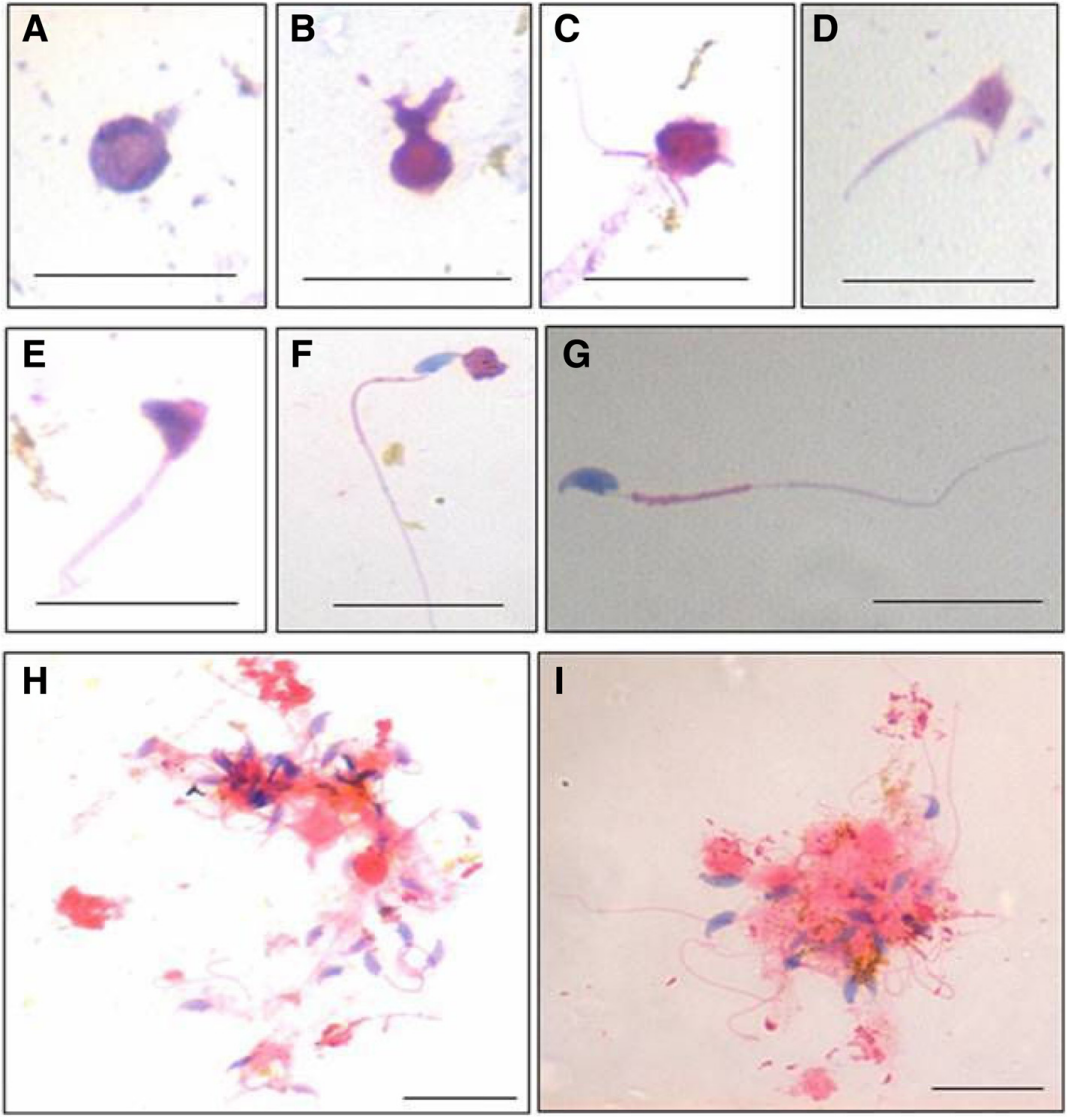
Hematoxylin and Eosin stained smears on Day 21. **(A-G)** Various stages of spermiogenesis were evident as described in literature [42]. Round spermatocytes with protruding tail, spermatid, elongated spermatid with prominent tail, with residual cytoplasmic bodies, fully mature sperm with a long tail, hook shaped head and prominent mid-piece were observed. **(H-I)** At places sperm were observed in clusters with their heads embedded in residual cytoplasmic body.

The cells (VSELs, possibly few spermatogonial stem cells and Sertoli cells) were studied after overnight seeding and two distinct populations of cells comprising the bigger Sertoli cells and distinctly spherical stem cells could be observed at lower magnification (Figure 1A). Sertoli cells were attached to the culture surface whereas the stem cells were not attached. At higher magnification (Figure 1B), two different size of stem cells were clearly visible including the smaller VSELs and slightly bigger SSCs. At places asymmetric cell division of VSELs giving rise to the SSCs could be observed (broken circles, Figure 1B&E). SSCs appeared to undergo rapid divisions, formed chains with incomplete cytokinesis (arrow, Figure 1B-E) and by days 3-7, clusters of germ cells were observed (Figure 1F&G) possibly reflecting clonal expansion of SSCs with incomplete cytokinesis. The germ cells were often observed in close association with Sertoli cells which were attached and differentiated into large mesenchymal-like fibroblasts (Figure 1C-E). The close association of differentiating germ cells with the mesenchymal cells was remarkable and observed on Day 7 also (Figure 2A) and possibly the somatic cells provided the ‘niche’ (source of growth factors) for germ cells differentiation. Similar close association of germ cells with mesenchymal cells has been observed during ovarian cultures also [26-28]. By day 10- 21 we observed germ cells in different stages of differentiation - implying that it is not a synchronous culture that we have developed -rather cells are in different stage of differentiation. Round to elongated spermatid undergoing various stages of spermiogenesis i.e. transition of spermatid into sperm was clearly visualized by Day 21 in culture (Figure 2C) under stereo microscope and after H & E staining (Figure 3). Elongated spermatid with cytoplasm covering the sperm head and part of tail were also visualized (Figure 3D-G). Excess cytoplasm was eventually lost as residual bodies (Figure 3H). Mature spermatozoa with characteristic hook shaped head and a distinct mid piece were visualized easily. It was observed that the sperm existed singly as well as in small clusters (Figure 3) with pink-stained cytoplasm covering the mid region. The cytoplasmic lobes appeared fused at places and are described as ‘residual bodies' in literature. Sperm showed certain degree of motility in the culture (Additional files 2 and 3).

**Figure 4:**
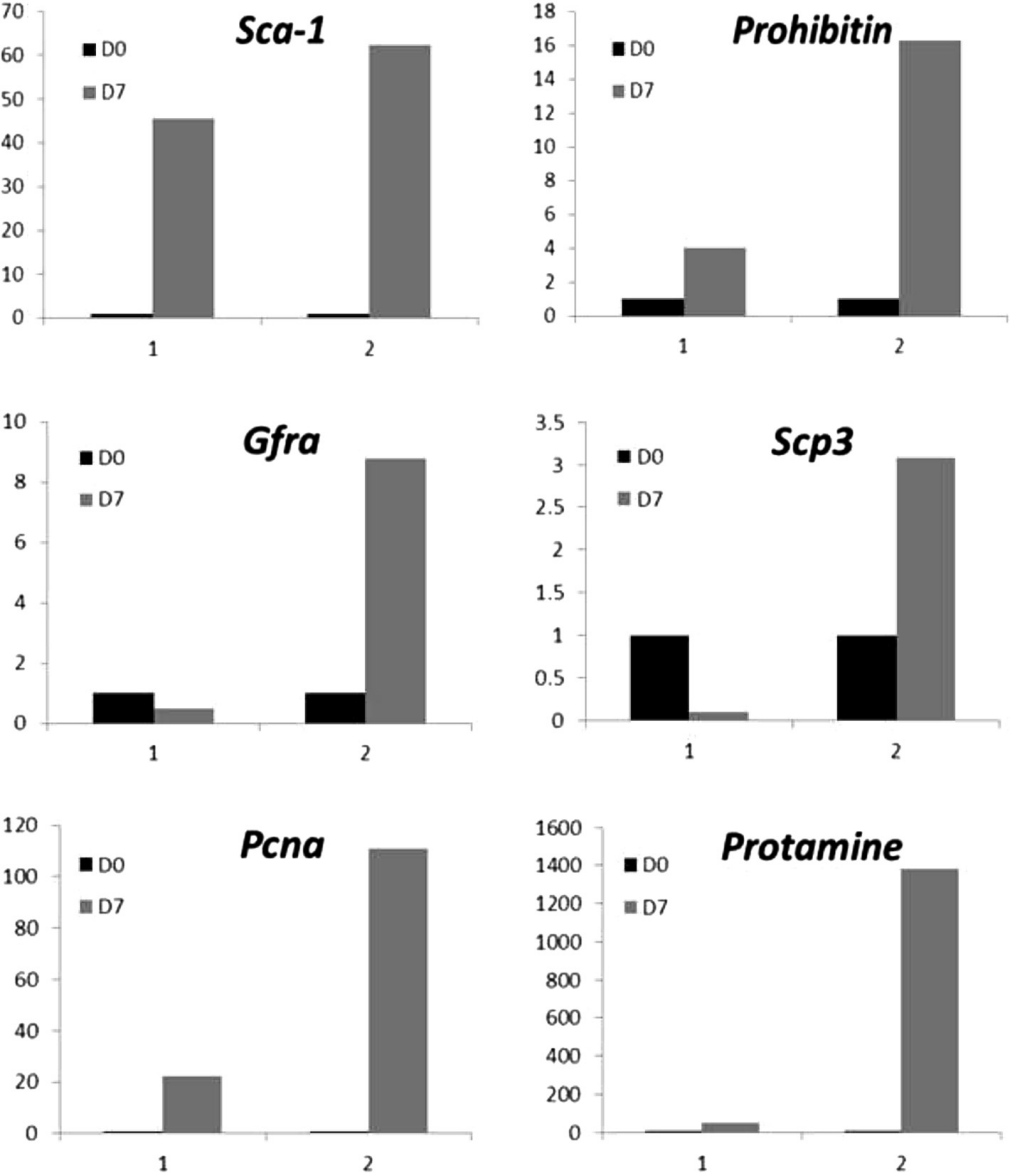
Quantitative RT-PCR data from two independent cultures on D7 compared to cells used for seeding (D0). An upregulation was noted for various transcripts but apparently the response in the cultures differed. An upregulation (> 40 folds) of Sca-1 in both the cultures was suggestive of self-renewal of VSELs in culture as Sca-1 is a marker for mouse VSELs [11]. An increase in Gfra (marker specific for spermatogonial stem cells) suggested proliferation of spermatogonial stem cells. Increased proliferative activity in VSELs as well as SSCs was confirmed even by upregulation of Pcna in both the cultures. Cells in the culture underwent meiosis as Scp3 and prohibitin (spermatocytes specific marker) transcript were upregulated *in vitro*. Protamine (post-meiotic marker for spermatid and sperm) transcript was also found upregulated suggesting differentiation of sperm in both the cultures (>1200 folds in one culture and > 40 folds in another). Variability in extent of upregulation of various transcripts during spontaneous differentiation of stem cells into sperm in the two cultures reflects biological variability and different stage of differentiation when cells were harvested for RNA extraction. However, in both cultures proliferation of cells and differentiation into sperm occurred.

Cells were harvested 7 days after seeding to evaluate progression of spermatogenesis by RT-PCR. *Gfra, prohibitin and protamine* were found to be expressed on day 7 (Figure 2, bottom panel). Quantitative RT-PCR analysis to study transcripts specific for stem cells (*Sca-1, Gfra*), proliferation (*Pcna*), meiosis (*Scp3*), pre-meiotic stage (*prohibitin*) and post-meiotic sperm (*protamine*) were repeated twice and results are represented individually for both the experiments (Figure 4). A distinct up regulation of various transcripts suggest the proliferation of stem cells and their progression/differentiation (spermatogenesis as well as spermiogenesis) into sperm *in vitro*.

## Discussion

Present study demonstrates for the first time that testicular cells (enriched for quiescent VSELs) which survive busulphan treatment in adult mice testis undergo spontaneous proliferation, clonal expansion, differentiation and meiosis resulting in sperm formation during *in vitro* culture for 3 weeks. We reported similar ability of human, sheep, monkey and rabbit ovarian VSELs enriched in ovary surface epithelium in 2011 [27] and recently in mouse ovary [26] to differentiate spontaneously into oocyte-like structures *in vitro*. Remarkable feature of the present study is that no additional growth factors were added to support differentiation and meiosis of germ cells. Rather conditioned medium from Sertoli cells culture was used and Sertoli cells that survive busulphan treatment attach to the bottom of the dish and provide a somatic support to the differentiating stem cells. It was difficult to make any quantification or ascertain the efficiency of the process of *in vitro* spermatogenesis implying how many VSELs/SSCs can give rise to how many sperm- rather the present study is more of an observational study and that all the 5 times the cultures were set up - sperm were invariably obtained at the end.

Although VSELs exist as a small sub-population comprising approximately 0.03% cells in normal testis, the numbers almost double after busulphan treatment [14]. In culture, VSELs underwent asymmetric cell division to give rise to the SSCs which divided in a symmetric manner and increased in numbers as chains and underwent clonal expansion to form clusters in agreement with published data in human testis [15]. Thus it is the true pluripotent stem cell state of VSELs which accounts for the remarkable differentiation observed in the present study. The increased numbers of VSELs and SSCs *in vitro* is further supported by more than 40-60 folds up-regulation of *Sca-1 *and also around 8 fold increase in *Gfra t*ranscripts by qRT-PCR. Elevated levels of *Sca-1* in both the culture samples analyzed by qRT-PCR was associated with low level of *Gfra* expression in one of the sample and this perhaps represents different state of proliferation/differentiation of the cells in culture taken for RNA extraction.

Whether a small population of SSCs survives after treatment depends on the dose of busulphan used. They may survive because this could only explain the spontaneous and endogenous recovery later on in certain cases. The concept that VSELs exist in testes, survive because of their quiescent status and may be responsible for endogenous recovery of spermatogonial cells are novel concepts in testicular biology put forth for the first time by our group. Similarly VSELs survive in adult mouse ovary after chemotherapy and are responsible for endogenous recovery of oocytes [26]. Choi et al [29] discussed that a sub-population of SSCs may be quiescent and escape damage by busulphan treatment whereas Zohni et al [30] reported that more than 95% of SSCs are destroyed within three days of busulphan treatment (irrespective of the dose) and the recovery observed after 10-12 weeks depends on the dose of busulphan. At four weeks after busulphan treatment, when cells were collected to establish cultures, SSCs remain undetectable. Thus over time it is the VSELs which may have given rise to SSCs to restore testicular function, but remained undetected till date because of their very small size. There is no need to compare or deliberate on the potential of VSELs and SSCs to give rise to sperm *in vitro* because VSELs invariably will give rise to SSCs which will further differentiate into sperm. The concept that VSELs give rise to SSCs was proposed by us [16] based on OCT-4 staining pattern observed while studying human testicular tissue. Being a transcription factor, OCT-4 acts by binding to DNA and regulates the expression of several genes. It is crucial for pluripotency & self-renewal and silencing OCT-4 results in differentiation of ES cells [31]. Similar OCT-4 isoforms are described in ovary by others also [32]. Being pluripotent VSELs express nuclear OCT-4 which is no longer required to maintain the pluripotent state, shifts to the cytoplasm in the immediate descendants SSCs and eventually disappears upon further differentiation.

Correct meiotic progression *in vitro* has remained the major bottle-neck and various groups have used chemicals like retinoic acid for meiotic entry of germ cells [33]. The successful results in the present study are attributed to the healthy niche/physical support provided by the attached Sertoli cells to the developing germ cells and use of conditioned medium from healthy Sertoli cells. Similar differentiation potential of ovarian VSELs lodged in the ovary surface epithelium into oocyte-like structures *in vitro* was reported earlier by our group [26-28]. Thus the testicular cells cultures in the present study were established with a conviction and we wanted to ascertain whether testicular VSELs behave the same way as ovarian VSELs.

Considerable progress has been made by various groups to demonstrate spermatogenesis *in vitro* and was recently reviewed [2,4,34]. Sato et al [35] produced functional sperm from neonatal mouse testes using an organ culture method and also produced healthy and reproductively competent offspring using spermatid and sperm for micro-insemination. Abu Elhija et al [36] reported that germ cells from 7 days old immature mice can be expanded and differentiated in a soft agar 3D culture to produce morphologically normal sperm. The importance of microenvironment ‘niche’ to achieve stem cells proliferation/ differentiation has gained considerable importance [37] and also in the field of testicular stem cells and thus a thrust on use of 3D culture in place of conventional 2D culture in Petri dish [34,38]. However, as recently concluded by Stukenborg’s group [34] much more remains to be achieved to successfully achieve meiosis *in vitro* since the support provided by Sertoli cells and peritubular cells *in vivo* could not be replicated *in vitro* and also even though several combinations of culture media were tried, none was competent enough to support complete spermatogenesis.

The Views and Reviews section in Fertility Sterility published in January 2014 concludes that the field of obtaining gametes from pluripotent stem cells remains a distant dream and much more remains to be achieved on the bench before moving to the clinic [1]. The remarkable ability of VSELs to differentiate into gametes *in vitro*, unlike the ES/iPS cells lies in their source of origin during development and was recently discussed by us in details. It is the distinct epigenetic status of the VSELs and their close similarity to PGCs confers on them the superior ability to spontaneously differentiate into sperm compared to ES/iPS cells [39]. Recently Surani's group reported SOX 17 as an important specifier to convert ES/iPS cells into PGCs [40]. But VSELs are indeed PGCs that survive in small numbers in adult gonads and thus may prove to be ideal endogenous pluripotent stem cell candidates to restore fertility in cancer survivors. They survive oncotherapy and thus there may be no need to cryopreserve the testicular tissue from pre-pubertal boys who cannot provide sperm for banking. Our studies have shown both *in vitro* (present study) and *in vivo* [15] potential of VSELs to undergo spermatogenesis. The sperm produced after improving the niche in chemoablated testis [15], traverse the epididymis and accumulate in large numbers in the cauda after cell therapy compared to vehicle transplanted group, show normal motility and also have potential to fertilize the oocytes *in vitro*. Similar experiments need to be urgently undertaken using human testicular biopsies from azoospermic cancer survivors. Differences exist between mice and men and the question remains whether our work in mice has relevance to humans. Our clinical collaborators have observed similar VSELs in adult azoospermic survivors of childhood cancer patients (unpublished data of Dr Purna Kurkure at Tata Memorial Hospital, Mumbai). We are not advocating obtaining sperm *in vitro* for clinical use. We will rather take advantage of Mother Nature and prefer to restore spermatogenesis by providing a healthy niche *in situ* as described in our recent study [15]. Obtaining sperm *in vitro* will always have associated epigenetic concerns and results of the present study only demonstrate the differentiation potential of VSELs to the scientific community.

The scientific community needs to take note of this novel population of pluripotent stem cells that exist in adult testis and exploit their potential. Importance of a healthy niche/ microenvironment also needs to be appreciated. Several decades of research targeted the ‘seeds’ for fertility preservation; little realizing that it is the soil that needs to be rejuvenated. The results of present study and our recently published studies [15,26] imply that endogenous VSELs that survive oncotherapy could be exploited to restore normal fertility in cancer survivors. However, studies in higher animal models and pilot clinical studies are warranted before extrapolating these results to clinics. In addition to cryopreserving sperm and testicular biopsy as a source of germ cells; Sertoli cells should be collected, expanded and stored prior to oncotherapy. The Sertoli cells may be later injected through inter-tubular route as a simple OPD procedure hopefully to restore normal fertility. As an alternative mesenchymal cells (from autologus adipose tissue or bone marrow) could also benefit existing azoospermic survivors who were deprived of sperm banking prior to therapy. Our results provide a paradigm shift, have translational potential and provide alternate options which may prove to be more practical and affordable than currently available options to achieve biological parenthood in cancer survivors. Further studies are required to be undertaken by independent groups to confirm existence of nuclear OCT-4 expressing VSELs in adult testis and ovaries and their potential to regenerate chemoablated gonads. It is the pluripotent primordial germ cells (express nuclear OCT-4) that migrate into the gonadal ridge and give rise to germ cells during early development possibly survive in adult gonads as VSELs. It was just providing the correct niche that resulted in successful stem cell biology *in vitro* in the present study. Importance of the niche was highlighted in a focus issue on Regenerative Medicine recently published in the journal Nature Medicine [41].

## Conclusions

To conclude, it is possible to coax VSELs that survive busulphan treatment in mice testis to undergo spermatogenesis *in vitro*. This better potential of VSELs compared to ES/iPS cells is because they are the PGCs surviving in adult testis. PGCs are more mature and pre-programmed to easily differentiate into gametes compared to inner cell mass cells in a blastocyst from which ES cells are derived. However, for clinical applications we advocate to restore spermatogenesis by providing a healthy niche *in vivo* as reported earlier [15] rather than *in vitro* maturation. The *in vivo* re-initiation of spermatogenesis by injecting healthy niche cells may provide normal fertility and biological parenthood to cancer survivors with minimal safety, ethical, regulatory and societal concerns.

## Additional files

Additional file 1:
**Supplemental Article file S1.** Very small embryonic-like stem cells survive and restore spermatogenesis after busulphan treatment in mouse testis. Full text of reference number 14. Additional file 2:
**Supplemental Video S1.** Dynamic nature of culture after 3 weeks showing subtle movement of sperm with hook-shaped head and well-defined tail. Additional file 3:
**Supplemental Video S2.** Another field of culture showing sperm after 3 weeks of culture.

## Abbreviations

VSELs: Very small embryonic like stem cells
PGCs: Primordial germ cells
ES cells: Embryonic stem cells
iPS cells: Induced pluripotent stem cells
EG cells: Embryonic germ cells
SSCs: Spermatogonial stem cells
MSCs: Mesenchymal stem cells

## References

[1] Legro RS, Adashi EY (2014). Introduction: Germline stem cell therapy in humans: two are not enough. Fert Steril.

[2] Hou J, Yang S, Yang H, Liu Y, Liu Y, Hai Y, Chen Z (2014). Generation of male differentiated germ cells from various types of stem cells. Reproduction.

[3] Hanna CB, Hennebold JD (2014). Ovarian germline stem cells: an unlimited source of oocytes?. Fert Steril.

[4] Valli H, Phillips BT, Shetty G, Byrne JA, Clark AT, Meistrich ML, Orwig KE (2014). Germline stem cells: toward the regeneration of spermatogenesis. Fert Steril.

[5] Medrano JV, Pera RA, Simón C (2013). Germ cell differentiation from pluripotent cells. Sem Reprod Med.

[6] Hübner K, Fuhrmann G, Christenson LK, Kehler J, Reinbold R, De La Fuente R, Wood J (2003). Derivation of oocytes from mouse embryonic stem cells. Science.

[7] Nayernia K, Nolte J, Michelmann HW, Lee JH, Rathsack K, Drusenheimer N (2006). In vitro-differentiated embryonic stem cells give rise to male gametes that can generate offspring mice. Dev Cell.

[8] Kee K, Angeles VT, Flores M, Nguyen HN, Reijo Pera RA, Human DAZL (2009). DAZ and BOULE genes modulate primordial germ-cell and haploid gamete formation. Nature.

[9] Panula S, Medrano JV, Kee K, Bergström R, Nguyen HN, Byers B, Wilson KD (2011). Human germ cell differentiation from fetal- and adult-derived induced pluripotent stem cells. Hum Mol Genet.

[10] Eguizabal C, Montserrat N, Vassena R, Barragan M, Garreta E, Garcia-Quevedo L, Vidal F (2011). Complete meiosis from human induced pluripotent stem cells. Stem Cells.

[11] Zuba-Surma EK, Kucia M, Wu W, Klich I, Lillard JW, Ratajczak J, Ratajczak MZ (2008). Very small embryonic-like stem cells are present in adult murine organs: image stream-based morphological analysis and distribution studies. Cytometry Part A.

[12] Bhartiya D, Unni S, Parte S, Anand S (2013). Very small embryonic-like stem cells: implications in reproductive biology. Bio Med Res Internatl.

[13] Bhartiya D, Parte S, Patel H, Anand S, Sriraman K, Gunjal P, Mariusz R (2014). Pluripotent very small embryonic-like stem cells in adult mammalian gonads. Adult Stem Cell Therapies: Alternatives to Plasticity.

[14] Anand S, Bhartiya D, Sriraman K, Patel H, Manjramkar DD (2014). Very small embryonic-like stem cells survive and restore spermatogenesis after busulphan treatment in mouse testis. J Stem Cell Res Ther.

[15] Bhartiya D, Kasiviswanathan S, Unni SK, Pethe P, Dhabalia JV, Patwardhan S, Tongaonkar HB (2010). Newer insights into premeiotic development of germ cells in adult human testis using Oct-4 as a stem cell marker. J Histochem Cytochem.

[16] Shin DM, Zuba-Surma EK, Wu W, Ratajczak J, Wysoczynski M, Ratajczak MZ, Kucia M (2009). Novel epigenetic mechanisms that control pluripotency and quiescence of adult bone marrow-derived Oct4(+) very small embryonic-like stem cells. Leukemia.

[17] Kassmer SH, Krause DS (2013). Very small embryonic-like cells: biology and function of these potential endogenous pluripotent stem cells in adult tissues. Mol Reprod Dev.

[18] Grymula K, Tarnowski M, Piotrowska K, Suszynska M, Mierzejewska K, Borkowska S, Fiedorowicz K (2014). Evidence that the population of quiescent bone marrow-residing very small embryonic/epiblast-like stem cells (VSELs) expands in response to neurotoxic treatment. J Cell Mol Med.

[19] Johnson J, Bagley J, Skaznik-Wikiel M, Lee HJ, Adams GB, Niikura Y, Tschudy KS (2005). Oocyte generation in adult mammalian ovaries by putative germ cells in bone marrow and peripheral blood. Cell.

[20] Ratajczak MZ, Shin DM, Liu R, Marlicz W, Tarnowski M, Ratajczak J, Kucia M (2010). Epiblast/germ line hypothesis of cancer development revisited: lesson from the presence of Oct-4+ cells in adult tissues. Stem Cell Rev.

[21] Bhartiya D, Singh J (2014). FSH-FSHR3-Stem cells in ovary surface epithelium: basis for adult ovarian biology, failure, aging and cancer. Reproduction.

[22] Jones TD, Ulbright TM, Eble JN, Cheng L (2004). OCT4: a sensitive and specific biomarker for intratubular germ cell neoplasia of the testis. Clin Cancer Res.

[23] Jones TD, Ulbright TM, Eble JN, Baldridge LA, Cheng L (2004). OCT4 staining in testicular tumors: a sensitive and specific marker for seminoma and embryonal carcinoma. Am J Surg Pathol.

[24] Bhartiya D, Kasiviswananthan S, Shaikh A (2012). Cellular origin of testis-derived pluripotent stem cells: a case for very small embryonic-like stem cells. Stem Cells Dev.

[25] Ratajczak J, Wysoczynski M, Zuba-Surma E, Wan W, Kucia M, Yoder MC, Ratajczak MZ (2011). Adult murine bone marrow-derived very small embryonic-like stem cells differentiate into the hematopoietic lineage after coculture over OP9 stromal cells. Experimental Hematol.

[26] Sriraman K, Bhartiya D, Anand S, Bhutda S. Mouse ovarian very small embryonic-like stem cells resist chemotherapy and retain ability to initiate oocyte-specific differentiation. Reprod Sci. 2015;20. doi:10.1177/1933719115576727. 10.1177/1933719115576727 PMC4565482 25779995

[27] Parte S, Bhartiya D, Telang J, Daithankar V, Salvi V, Zaveri K (2011). Detection, characterization, and spontaneous differentiation in vitro of very small embryonic-like putative stem cells in adult mammalian ovary. Stem Cells Dev.

[28] Parte S, Bhartiya D, Patel H, Daithankar V, Chauhan A, Zaveri K, Hinduja I (2014). Dynamics associated with spontaneous differentiation of ovarian stem cells in vitro. J Ov Res.

[29] Choi YJ, Ok DW, Kwon DN, Chung JI, Kim HC, Yeo SM, Kim T (2004). Murine male germ cell apoptosis induced by busulfan treatment correlates with loss of c-kit-expression in a Fas/FasL- and p53-independent manner. FEBS Lett.

[30] Zohni K, Zhang X, Tan SL, Chan P, Nagano MC (2012). The efficiency of male fertility restoration is dependent on the recovery kinetics of spermatogonial stem cells after cytotoxic treatment with busulfan in mice. Hum Reprod.

[31] Schoorlemmer J, Jonk L, Sanbing S, van Puijenbroek A, Feijen A, Kruijer W (1995). Regulation of Oct-4 gene expression during differentiation of EC cells. Mol Biol Rep.

[32] Samardzija C, Quinn M, Findlay JK, Ahmed N (2012). Attributes of Oct4 in stem cell biology: perspectives on cancer stem cells of the ovary. J Ovarian Res.

[33] Geijsen N, Horoschak M, Kim K, Gribnau J, Eggan K, Daley GQ (2004). Derivation of embryonic germ cells and male gametes from embryonic stem cells. Nature.

[34] Reda A, Hou M, Landreh L, Kjartansdóttir KR, Svechnikov K, Söder O, Stukenborg JB (2014). In vitro spermatogenesis - optimal culture conditions for testicular cell survival, germ cell differentiation, and steroidogenesis in rats. Frontiers Endocrinol.

[35] Sato T, Katagiri K, Yokonishi T, Kubota Y, Inoue K, Ogonuki N (2011). In vitro production of fertile sperm from murine spermatogonial stem cell lines. Nature Commun.

[36] Abu Elhija M, Lunenfeld E, Schlatt S, Huleihel M (2012). Differentiation of murine male germ cells to spermatozoa in a soft agar culture system. Asian J Androl.

[37] Dimmeler S, Ding S, Rando TA, Trounson A (2014). Translational strategies and challenges in regenerative medicine. Nature Med.

[38] Stukenborg JB, Schlatt S, Simoni M, Yeung CH, Elhija MA, Luetjens CM (2009). New horizons for in vitro spermatogenesis? An update on novel three-dimensional culture systems as tools for meiotic and post-meiotic differentiation of testicular germ cells. Mol Hum Reprod.

[39] Bhartiya D, Hinduja I, Patel H, Bhilawadikar R (2014). Making gametes from pluripotent stem cells – a promising role for very small embryonic-like stem cells. Reprod Biol Endocrinol.

[40] Irie N, Weinberger L, Tang WW, Kobayashi T, Viukov S, Manor YS (2015). SOX17 is a critical specifier of human primordial germ cell fate. Cell.

[41] Editorial (2014). Advancing regenerative medicine. Nat Med.

[42] Hess RA, Renato de Franca L (2008). Spermatogenesis and cycle of the seminiferous epithelium. Adv Exptl Med Biol.

