# Supplementary material for: Chemoablated mouse seminiferous tubular cells enriched for very small embryonic-like stem cells undergo spontaneous spermatogenesis in vitro
Source: Reprod Biol Endocrinol. 2015 Apr 18;13:33. doi: 10.1186/s12958-015-0031-2 (PMC4407302; doi:10.1186/s12958-015-0031-2)
Supplement: Additional file 1: — Supplemental Article file S1. Very small embryonic-like stem cells survive and restore spermatogenesis after busulphan treatment in mouse testis. Full text of reference number 14. [file 12958_2015_31_MOESM1_ESM.pdf]

Stem Cell
Research & Therapy

Anand et al., J Stem Cell Res Ther 2014, 4:7
http://dx.doi.org/10.4172/2157-7633.1000216

Research Article Open Access

Very Small Embryonic-Like Stem Cells Survive and Restore Spermatogenesis after Busulphan Treatment in Mouse Testis

Sandhya Anand, Deepa Bhartiya*, Kalpana Sriraman, Hiren Patel and Manjramkar DD

Stem Cell Biology Department, National Institute for Research in Reproductive Health (NIRRH), Mumbai

Abstract

Study Objectives: Adult mammalian testes harbor a novel population of quiescent, pluripotent, very small embryonic-like stem cells (VSELs) along with spermatogonial stem cells (SSCs). Present study was undertaken to (i) characterize testicular VSELs (ii) investigate differential effect of chemotherapy on VSELs and SSCs and (iii) to restore the differentiation ability of surviving VSELs by providing healthy somatic microenvironment.

Methods: Effect of busulphan (25 mg/Kg) was studied on mouse testes. Syngenic Sertoli cells (10^5 cells per testis) and bone marrow derived mesenchymal cells (10^4 cells per testis) were transplanted separately through inter-tubular route. Effect of niche reconstruction was studied two months later by histology and immuno-localization of germ cell markers MVH and PCNA. Caudal sperm were evaluated for their ability to fertilize oocytes *in vitro*.

Results: VSELs were 2-6 µm in size, SCA-1$^+$/CD45$^-$/LIN$^-$, had high nucleo-cytoplasmic ratio and comprised 0.03% of testicular cells whereas SSC specific marker GFRa localized on a distinct, larger cell population. Busulphan selectively destroyed SSCs and other germ cells however, nuclear OCT-4A, Nanog, Sox-2 and SCA-1 positive VSELs (0.06%) survived. Persisting VSELs were unable to differentiate possibly because chemotherapy also affected the 'niche' comprising Sertoli cells. Complete restoration of spermatogenesis was observed two months post transplantation of Sertoli and mesenchymal cells. Transplanted cells formed neo-tubules in the vicinity of surviving tubules and were possibly a source of growth factors essential for VSELs proliferation and differentiation. Both MVH and PCNA showed increased staining in the transplanted group. qRT-PCR studies revealed existence of a meiotic block in busulphan treated testis which was overcome after transplantation. Resulting sperm progressed to epididymis, showed normal motility and ability to fertilize *in vitro*.

Conclusion: Results show that VSELs survive chemotherapy and can restore spermatogenesis in germ cells depleted mice. Results have direct relevance to address fertility issues of cancer survivors.

Keywords: Spermatogenesis; VSELs; Busulphan; Stem cells; Transplantation; Sertoli cells; Mesenchymal cells; Oncofertility

Introduction

The continuity of spermatogenesis throughout life in an adult mammal is facilitated by stem cells. Our group has earlier reported that adult human and mouse testes harbor two distinct stem cell populations that include quiescent very small ES-like stem cells (VSELs) with pluripotent characteristics and their active 'descendants' progenitors viz. spermatogonial stem cells (SSCs) [1,2]. Similar existence of two stem cell populations including VSELs and the progenitors (ovary germ stem cells, OGSCs) were previously identified in mammalian ovaries by our group [3,4]. Co-existence of quiescent and active stem cell populations is an emerging concept reported in other adult body tissues as well [5-7]. The two stem cell populations in the gonads are distinguished by their ability to differentially express OCT-4A [1,3], transcription factor known to be involved in maintenance of pluripotency in stem cells [8]. OCT-4 has two major isoforms namely OCT-4A, localized in the nucleus and essential for pluripotency and OCT-4B localized in the cytoplasm and whose biological function is not yet known [9,10]. An indiscriminate detection of Oct-4 and not the precise transcripts has led to lot of confusion in the literature [11,12]. VSELs express nuclear OCT-4A while their immediate descendants 'progenitors' express cytoplasmic OCT-4B [1,3]. Thus, based on two stem cell hypothesis, we presume that VSELs are 'quiescent' stem cells that serve as a backup pool, undergo asymmetric cell divisions to self-renew and give rise to progenitors which divide rapidly and further differentiate into tissue-specific cell types. VSELs thus may be required to maintain tissue homeostasis similar to that seen in other body tissues [13,14].

It is indeed intriguing as to why testicular VSELs have eluded reproductive biologists so far. We discussed in our earlier paper that this could be because of technical reasons [1]. Nevertheless, many reports are available that describe the presence of a sub-population of cells with pluripotent characteristics among SSCs in both mice and humans. Izadyar et al. [15] reported a subpopulation of SSCs with pluripotent SSEA4$^+$phenotype that has the ability to repopulate germ cell depleted testis in mice. Lim et al. [16] performed co-localization studies for GFRα-1 and SSEA-4 in spermatogonial stem cell cultured colony and reported that these markers representing germ and pluripotent stem cell do not co-localize, but exist as two distinct populations. Ratajczak et al. [17] were first to report the presence

***Corresponding author:** Dr. Deepa Bhartiya, Stem Cell Biology Department, National Institute for Research in Reproductive Health, Mumbai, India, Tel: +91 022 2419 2012; Fax: +91 022 2413 9412; E-mail: dbhartiya@nirrh.res.in

Received May 29, 2014; **Accepted** July 17, 2014; **Published** July 19, 2014

Citation: Anand S, Bhartiya D, Sriraman K, Patel H, Manjramkar DD (2014) Very Small Embryonic-Like Stem Cells Survive and Restore Spermatogenesis after Busulphan Treatment in Mouse Testis. J Stem Cell Res Ther 4: 216. doi:10.4172/2157-7633.1000216

Copyright: © 2014 Anand S, et al. This is an open-access article distributed under the terms of the Creative Commons Attribution License, which permits unrestricted use, distribution, and reproduction in any medium, provided the original author and source are credited.

of pluripotent VSELs in adult mice testis by flow cytometry. Several groups have reported the presence of a 'side population' with stem cell characteristics (having a small size and a low scatter) in testicular tissue of cockerels and mice [18-20]. These side population (SP) cells have the ability to repopulate germ cell depleted testis when transplanted in adult mice. Kubota et al. [21] reported that SSCs and SP in testis are two distinct populations; SSCs are not enriched in the testis SP, while the SP comprised of SCA$^+$ cells. Existence of testicular SSC subsets with label-retaining properties has also been reported by Grisanti et al. [22]. They reported previously unrecognized cell heterogeneity in murine A_s spermatogonial *in vivo.* Our group recently reviewed the presence of VSELs in mammalian gonads [23]. VSELs have also been hypothesized to be the embryonic remnants responsible for various cancers [24,25]. It is also interesting to point out here that OCT-4 is one of the sensitive markers increasingly being used for diagnosis of carcinoma *in situ* in germ cell tumors [25,26]. Several groups have suggested that [27,28], these VSELs may be the similar to the MAPCs described by Verfaillie's group [29]. Many groups have reported successful derivation of ES-like colonies from testicular tissue culture in mice [30] as well as in humans [31,32] and proposed the origin of the colonies to be dedifferentiation of SSCs. However, several lines of evidence suggest pluripotent stem cells (VSELs) present in testicular tissue may actually be giving rise to these ES-like colonies [2].

Interestingly the two stem cell populations in testis differ in their proliferation kinetics; VSELs are quiescent and probably divide rarely while the progenitors (SSCs) exhibit spurts of active cell divisions as evident by the presence of chains of spermatogonia with cytoplasmic OCT-4 undergoing clonal expansion [1]. Present study was undertaken to characterize these stem cells in more detail and to study the effect of busulphan treatment on them in mouse testis, since chemotherapy targets actively dividing cells in the body along with tumor cells. We observed that VSELs persist in germ cell depleted mouse testis possibly due to their quiescent nature. The persisting VSELs were unable to differentiate and this could be due to the compromised niche comprising mainly of Sertoli cells as suggested earlier [33,34]. Sertoli cells are the main source of growth factors required for spermatogenesis to occur. Hence, the present study also investigated restoration of functionality of the persisting VSELs *in vivo* by transplanting neonatal Sertoli cells or bone marrow derived mesenchymal cells in busulphan treated mouse testis.

Materials and Methods

All experiments carried out in the present study were approved by Institutional Animal Ethics Committee. Eight weeks old adult male Swiss mice, maintained in the Institute experimental animal facility were used for the study. They were housed in a temperature and humidity controlled room on a 12 light/12 darkness hour cycle with free access to food and water. The mice (n=3 per group) were initially treated with four different doses of busulphan - (i) 25 mg/Kg body weight busulphan (ii) 15 mg/Kg busulphan and 100 mg/Kg cyclophosphamide (iii) 12 mg/Kg busulphan and 120 mg/Kg cyclophosphamide and (iv) 10 mg/Kg busulphan daily for 4 days and 100 mg/Kg cyclophosphamide on first 2 days; in order to select a dose that resulted in complete germ cell depletion Busulphan (Sigma-Aldrich, USA) was dissolved in DMSO (Sigma-Aldrich), diluted with equal volume of injection grade water and injected intra-peritoneally. Cyclophosphamide (Zydus, India) was dissolved in sterile injection grade water and also injected intra-peritoneally with a minimum 2 hours gap between the two injections. Control mice (n=5) were injected with vehicle alone. One month after the treatment, mice were sacrificed by cervical dislocation; testes were collected, weighed and further processed. Maximum germ cell loss was obtained with a dose of 25 mg/Kg busulphan and hence this dose was selected for all experiments performed in the present study.

Experimental details

Effect of busulphan treatment on spermatogenesis and testicular stem cells: Testicular tissue collected after one month of busulphan treatment was appropriately processed for histology, flow cytometry and RNA studies. Standard histology studies on testicular sections were done to study the effect of busulphan treatment on spermatogenesis. VSELs (Sca-1, Oct-4A) and germ cells (Oct-4, Gfra, and Deleted in Azoospermia-Like DAZL) markers were studied by qRT-PCR and immuno-localization to reveal differential effect of busulphan treatment on various cell populations. Flow cytometry and immunolocalization studies were also carried out to further quantitate and characterize SCA$^+$/CD45$^-$/LIN$^-$VSELs in normal and busulphan treated testis. Further, immuno-localization and immuno-phenotyping studies were carried out to show that the VSELs and spermatogonial stem cells (SSCs) are distinct stem cell populations in the testes using specific markers for VSELs (SCA-1) and SSCs (GFRa). Details of various methods are provided below.

Niche reconstruction in chemoablated testes: Once VSELs were found to persist in the testes despite complete loss of SSCs and germ cells in busulphan treated mice, we worked on the hypothesis that chemotherapy destroys actively dividing cells and the niche leading to lack of differentiation of VSELs in busulphan treated mouse testis. Attempts were made to restore testicular function by re-constructing the damaged niche in chemoablated testes by transplantation of healthy (i) Sertoli cells, since they form the natural niche in testis [34] and (ii) bone marrow derived mesenchymal cells as they are a rich source of various growth factors known to have beneficial effect in tissue regeneration [35]. All transplantations were done two months post busulphan treatment. Various experiments were conducted over a period of four months as shown in Figure 1.

Transplantation of Sertoli cells: The method we used is similar to published method described by Zhang et al. [36]. Sertoli cells were isolated from 8-10 days old neonatal mice testis for transplantation studies. Briefly, the testes were collected in sterile Dulbecco's Phosphate Buffer Saline (DPBS, Invitrogen, USA). After 2-3 washes with DPBS containing penicillin-streptomycin (Invitrogen), the testes were detunicated and chopped in media containing DMEM F12 with 20% fetal bovine serum (FBS, Invitrogen) and antibiotics. Cells were filtered through 100, 70 and 40 um cell strainers (BD Falcon; USA) and then plated into 60 mm culture dishes and incubated overnight at 37^{0}C in a humid 5% CO_2 environment. The Sertoli cells attach to the plates whereas the germ cells remained floating or loosely attached to Sertoli cells. The plates were rinsed and media changed to remove the floating germ cells. The attached Sertoli cells were allowed to grow confluent. Once confluent, the cells were trypsinized with 0.05% Trypsin-EDTA (Invitrogen) and collected in plain DMEM high glucose (DMEM HG, Invitrogen) to be used for transplantation. Approximately 10^5 cells (100 µl) were injected into each testis via interstitial route. Briefly, the cells were injected at 2-3 random sites into the testis through the skin. Control animals were injected with vehicle alone.

Transplantation of mesenchymal cells: Mesenchymal cells were obtained from bone marrow cells of four weeks old female Swiss mice using a published procedure [37]. Briefly, long bones of the leg were collected by sacrificing the mice and the marrow was flushed with MSC culture medium DMEM containing15% FBS, 1% non-essential

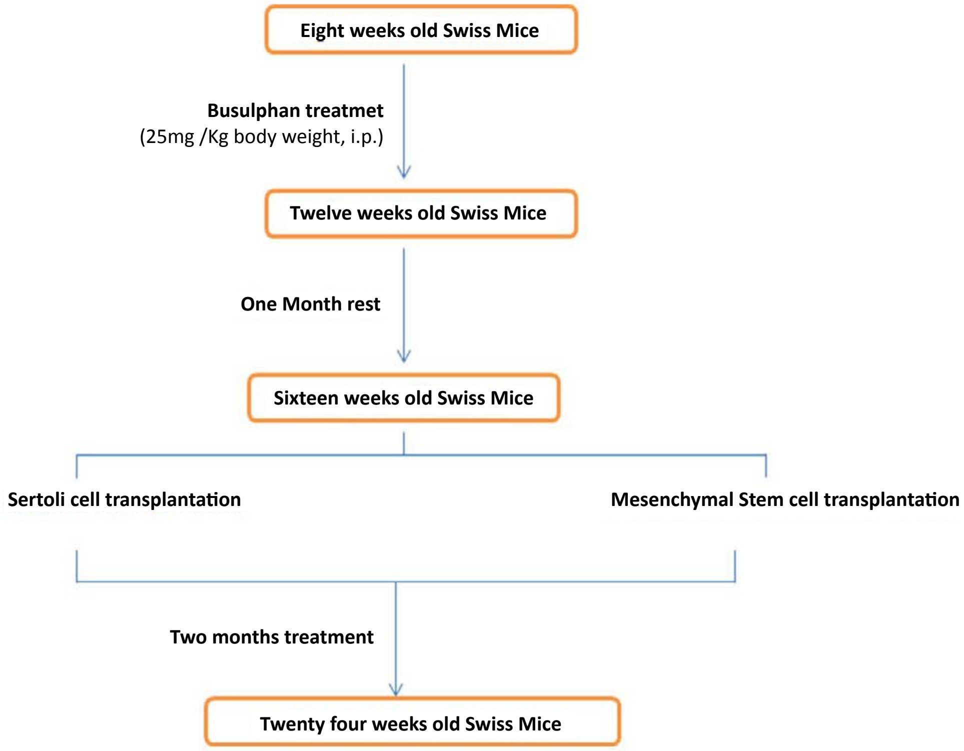

Figure 1: Treatment schedule and transplantation details. Eight weeks old mice are treated with busulphan and studied for testicular germ cell depletion after one month. The germ cell depleted mice are further rested for a month and then used for transplantation studies. They are sacrificed two months post- transplantation for various studies.

amino acids, 2 mM L-Glutamax, 0.1Mm β-mercapto-ethanol and 1% Penicillin- Streptomycin (Pen-strep) on ice. All media constituents were from Invitrogen. The marrow was resuspended with a Pasteur pipette to obtain single cell suspension and filtered through 70 µm cell strainer (BD Falcon), washed and plated in a six-well cell culture plate at a density of 5-6x10^6 cells per well and incubated at 37^0C, 5% CO_2 in a humid environment. Non-adherent cells were removed carefully after 3-4 hours and media was changed frequently up to 72 hours of initial culture. Further media changes were done every 3-4 days. Once culture attained about 70%-80% confluence, spindle shaped mesenchymal cells were isolated by controlled trypsinization with 0.25% Trypsin-EDTA at 25^0C for 2 minutes. The cells were re-plated in T-25 flasks and these P1 cells were used for transplantation upon confluency. For transplantation approximately 10^4-10^5 cells were injected by similar method described above for Sertoli cells. For control group, the busulphan treated mouse testis was injected only with medium used for resuspending cells (this group is named as vehicle transplanted/ control group).

Two months post transplantation; testes and epididymis were collected and processed to evaluate resumption of spermatogenesis by standard histology, morphometry, immunolocalization and qRT-PCR studies. H & E stained sections from various groups were studied to analyze effect of transplantation on restoration of spermatogenesis. Morphometric analysis was also carried out to understand the extent of spermatogenesis restoration. For this, tubules showing spermatogenesis and empty tubules were counted (expressed as percentage of total tubules counted) on a minimum of three sections from 3-5 animals in vehicle, Sertoli cells and mesenchymal cells transplanted groups. T-Test was used for calculating significance and $p<0.05$ was considered significant. Immuno-localization studies were carried out for testicular germ cell markers including (i) proliferating cell nuclear antigen (PCNA) specific for the stem cells and (ii) mouse vasa homolog (MVH), specific for spermatogonia and their immediate descendants. Quantitative RT-PCR studies were carried out in vehicle and Sertoli and mesenchymal cells transplanted groups using markers for VSELs (Sca-1), spermatogonial stem cells (Gfra), meiotic (prohibitin) and post-meiotic (protamine) markers.

Experiments using GFP mice were also conducted to demonstrate that the resumption of spermatogenesis was indeed from the persisting VSELs and not from the transplanted cells. For this Sertoli cells and mesenchymal cells were cultured using GFP mice (the FVB GFP mice strain [FVB.Cg-Tg(GFPU) 5NAGY/J] obtained from National Institute of Immunology, New Delhi) and transplanted into normal wild-type mice using methods described above. The sperm were directly viewed under confocal microscope for GFP expression.

Demonstration of in vitro fertilization potential of caudal sperm collected after testicular niche reconstruction: Four weeks old female Swiss mice were stimulated with 5IU of pregnant mare serum gonadotropin (National Hormone & Peptide Program, Harbor-UCLA Medical Center, USA) and 5IU of hCG (Ovirup, India) was given 48 hours after PMSG. The oocytes were collected after 12 hours of hCG injection in DMEM F12. The eggs were incubated for *in vitro* maturation for 3-4 hours at 37^0C under 5% CO_2 in DMEM F12 containing serum and growth factors namely EGF, hCG and FSH. Sperm were collected from the epididymis of transplanted mice in DMEMF12, their motility was recorded and added to the droplets with oocytes for *in vitro* fertilization in Sydney IVF fertilization medium (COOK,

Australia) and monitored for their ability to attach to the oocytes. The oocytes were then transferred to Sydney IVF cleavage media (COOK). Representative images were captured under inverted microscope (TE2000, NIKON, Japan). Further the oocytes were transferred to Sydney IVF blastocyst media (COOK) to check blastocyst formation.

Details of various methods used in the study

Preparation of single cell suspension from mouse testis: For testicular tissue from control and busulphan treated animals, single cell suspension was prepared as described elsewhere [1]. This suspension was used for both flow cytometry as well as for making smears to characterize the VSELs. Briefly, this involved detunication of testes, washing the tubules in phosphate buffer saline (PBS) followed by sequential enzymatic digestion with 1mg/ml collagenase IV (Invitrogen) and 0.25% trypsin EDTA and pipetting. The cell suspension was filtered through 40 µm cell strainer. The filtrate was used to make smears on poly-L-lysine coated slides and were fixed with 4% paraformaldehyde (Sigma) for 15 minutes, washed with PBS, air-dried, and stored for future use at 4^0C.

Histology: Testicular tissue was fixed in 10% neutral buffered formalin for histological studies. 5 µm thick sections of paraffin embedded testes were prepared and stained with Hematoxylin and Eosin (H & E) to study the tissue histo- architecture and the effect of busulphan treatment. The representative areas were photographed using Nikon 90i microscope (Nikon, Japan) and data recorded.

Immuno-localization studies: A polyclonal OCT-4 antibody (ab19857, Abcam, UK), raised against C terminus of human OCT-4, that could detect both the isoforms viz. OCT-4A and OCT-4B with distinct nuclear and cytoplasmic localization respectively was used in this study. The specificity of this antibody was confirmed previously using different antibodies and techniques [1]. The polyclonal DAZL antibody (Abcam) rose against the C-terminus of mouse DAZL was used as a germ cell marker. SCA-1 and GFRa (Sigma) antibodies were used to characterize the pluripotent VSELs and SSCs respectively. PCNA (Sigma) and MVH (R&D Biosystems, USA) antibodies were used to study the effect of transplantation on testicular germ cells. Immuno-localization studies by ICC, IF and IHC were repeated at least three times on three different samples.

(A) Immuno-histochemistry (IHC): NBF fixed tissue sections were used for IHC studies. Briefly the paraffin sections were deparaffinized and rehydrated through a graded methanol series. Endogenous peroxide was blocked using 0.3% hydrogen peroxide for 30 minutes in dark at room temperature. Antigen retrieval was done by treating the sections with sodium citrate buffer (10 mM sodium citrate), pH 6.0, at high power for 5 minutes in a microwave oven. Blocking was done with 10% normal goat serum for 1 hour, followed by incubation with the primary antibody at 4°C overnight. Primary antibody was replaced with blocking solution for negative control. After washes with PBS, the smears were incubated with the biotinylated secondary antibody for 30 minutes followed by Avidin Biotin Complex formation step for 30 minutes (Vectastain Elite ABC kit, Vector Laboratories Inc,USA), extensive wash with PBS and then detection was done using diaminobenzidene (Biogenex, USA) and counterstained with Haematoxylin. The sections were again dehydrated and mounted using DPX mountant (Qualigens, India). Representative areas were photographed under Nikon 90i microscope and the data was recorded.

(B) Immuno-cytochemistry (ICC): For ICC, the endogenous peroxide was blocked using 3% hydrogen peroxide for 30 minutes in dark at room temperature. Antigen retrieval was done by treating the smears with sodium citrate buffer (10 mM sodium citrate), pH 6.0, at high power for 5 minutes in a microwave oven. This was followed by permeablization step with 0.3% Triton-X 100 (Sigma) for 10 minutes. Further the smears were processed for immunolocalization in a manner similar to that described for IHC.

(C) Immunofluorescence (IF): The mouse testicular smears were washed with 0.5%BSA (Sigma) followed by blocking with 3% BSA for 1 hour. The cells were permeabilized using 0.3% Triton-X 100 for 5 mins and incubated with OCT-4A antibody (Millipore, USA) overnight at 4^0C. Following incubation of primary antibody, the smears were washed with wash buffer and incubated with fluorescent tagged secondary antibody (Alexafluor-488) for 2 hours at room temp in dark. The cells were washed thrice with wash buffer and counterstained with DAPI for 15 minutes (or PI for 2 minutes). Similarly, staining was performed using NANOG and SOX-2 antibodies. Dual staining of NANOGand STELLA was done with Alexafluor 488 as secondary for NANOG and Alexafluor 568 as secondary for STELLA. For cell surface markers, namely SCA-1-FITC, LIN-APC, CD45-PE (BD Biosciences, USA) and GFRa, the permeabilization step was omitted. The incubation of SCA-1-FITC was done for 2 hours at 4^0C. For co-localization studies with phalloidin, primary antibody incubation was followed by phalloidin-TRITC (Sigma) staining for 45 minutes. Co-localization of SCA-1-FITC, LIN-APC and CD45-PE was done by incubating with primary antibodies for 2 hours at 4^0C.

(D) Immunofluorescence on cryo-section of testicular tissue: Cryosections were used for triple staining of SCA-1, LIN and CD45 antibodies on mouse testicular VSELs. For this, the tissue was fixed in Tissue-tek OCT compound (MILES, USA) and frozen at -80^0C. Cryosections were cut on Leica CE (Germany) microtome. Further, the frozen sections were fixed with 4% PFA for 1 hour, washed with chilled acetone followed by PBS. This was followed by blocking and primary antibody incubation as described above for immunofluorescence on cell smears.

Flow cytometry studies: Flow cytometry studies were carried out to (i) quantitate the number of VSELs and (ii) for immuno-phenotyping studies. The testicular cell suspensions from control and busulphan treated mice prepared by enzymatic digestion as described above ,were washed and stained with SCA-1-FITC, LIN-APC and CD45-PE to quantitate the number of VSELs. Cells were acquired and analyzed on FACS Aria (BD) according to the gating strategy. Gates were set using unstained sample for each of the antibodies.T-Test was used for calculating significance and $p<0.05$ was considered significant. Testicular cell suspension from normal mice was stained with GFRa antibody for 2 hours at room temperature. Following washes to remove primary antibody, Alexaflour 568 conjugated secondary antibody was added for 1 hour at room temperature. FACS analysis was performed on FACS Aria. For antibody staining, gates were applied using unstained samples.

Quantitative RT-PCR studies: Testicular tissue was fixed in TRIzol (Invitrogen) for RNA isolation and stored at -80^0C till further processing. Total RNA was extracted using standard protocol using TRIzol and treated with DNase I (Fermentas, USA) to remove any genomic DNA present. First-strand cDNA was synthesized using the iScriptc DNA synthesis Kit (Bio-Rad, USA) according to the manufacturer's instructions. Briefly, 1µg of total RNA was incubated with 5x iScript reaction mix and reverse transcriptase mix. The reaction was carried out in G-STORM thermocycler (Gene Technologies, UK) as per manufacturer's instructions.

The expression levels of various gene transcripts namely total Oct-4, Oct-4A, Dazl, Gfra, Sca-1, Prohibitin and Protamine were estimated by CFX96 real-time PCR system (Bio-Rad Laboratories, USA) using SYBR Green chemistry (Bio-Rad). Gapdh was used as housekeeping in mice experiments. The primers used in the study are mentioned in Table 1. The amplification conditions were: initial denaturation at 94°C for 3 minutes followed by 40 cycles comprising of denaturation at 94°C for 10 seconds, annealing for 20 seconds, and extension at 72°C for 30 seconds followed by melt curve analysis. The fluorescence emitted was collected during the extension step of each cycle. The homogeneity of the PCR amplicons was verified by running the products on 2% agarose gels and also by studying the melt curve. All PCR amplifications were carried out in duplicate. Mean Ct values generated in each experiment using the CFX Manager software (Bio-Rad) were used to calculate the mRNA expression levels. Since ΔCt is inversely proportional to relative mRNA expression levels, the levels were calculated manually by the ΔCt method. The fold change was calculated using ΔΔCt method. The relative expression levels of each gene from at least 3 control mice and 3 treated mice were compared and the average value is reported. In all qRT-PCR experiments, the value of average fold change has been calculated with respect to busulphan treated animal. T-Test was used to calculate significance and $p<0.05$ was considered significant.

Results

Effect of busulphan treatment on testicular spermatogenesis and stem cells

Effect on spermatogenesis: Treatment of mice with busulphan reduced both the size and weight of the testes compared to the normal untreated testis (Figure 2A-2F). A well-organized pattern of spermatogenesis was observed in normal testis (Figure 2G) with the sperm arranged radially in the lumen of the tubule and prominent Sertoli cells with extensive cytoplasm spanning the whole cross-section of the tubule into the lumen wherein spermatogonial cells in different stages of differentiation are embedded. In contrast, the busulphan treated seminiferous tubules (Figure 2H) revealed extensive germ cell loss. Very few tubules showed some degree of spermatogenesis but majority were empty, devoid of spermatogonial cells and minimal haploid cell population was observed in all the mice (n=10 mice) studied during the course of the study. Sertoli cells appeared collapsed due to absence of germ cells. Interestingly the interstitial compartment comprising of Leydig cells, appeared much more prominent after busulphan treatment (Figure 2H). These observations are consistent with earlier reports that busulphan effectively destroys dividing cells and disrupts normal spermatogenesis.

Germ cell specific markers were used to confirm busulphan effect at both protein (DAZL) and mRNA (Dazl and Gfra) level by immunolocalization and qRT-PCR studies. Compared to the normal testis where DAZL (pre-meiotic germ cell specific marker) was localized in the cytoplasm of spermatogonial cells and spermatocytes lining the basement membrane of the seminiferous tubules (Figure 2I), busulphan treatment resulted in almost complete loss of DAZL positive cells in the seminiferous tubules (Figure 2J). This correlates well with complete reduction of Gfra transcript which is specific for SSCs ($p<0.05$) and Dazl ($p<0.01$) observed after busulphan treatment (Figure 2K). This data was obtained as mean ± SD from at least three independent experiments.

From these results, it was concluded that 25mg/Kg body weight of busulphan for germ cell depletion effectively destroyed germ cells including SSCs and hence this dose of busulphan was used for further characterization and niche reconstruction studies.

Characterization of stem cells (VSELs) that resist busulphan treatment: Stem cells (VSELs) that resisted busulphan treatment were characterized using pluripotent markers (OCT-4, SCA-1, NANOG, SOX-2) by techniques like immunolocalization, qRT-PCR and flow cytometry. Earlier immunolocalization studies reported by our group show that polyclonal OCT-4 antibody is differentially localized in the nucleus of pluripotent stem cells and in the cytoplasm of progenitors [1,2]. In this study, the busulphan treated testicular smears and sections showed only nuclear OCT-4A positive pluripotent stem cells whereas the cytoplasmic OCT-4 positive active progenitors were lacking (Figure 2C-F). There was no consistent pattern as the numbers of positive cells varied per tubule; some tubules did not have OCT-4A positive cells whereas others had few to several OCT-4A positive cells. We observed SCA-1^+cells localized close to the basement membrane, interspersed between Sertoli cells (Figure 3D). Triple staining was carried out on cryo-sections showed that SCA-1^+cells were LIN^- and $CD45^-$ (Figure 3E), described to be VSELs in literature [17]. They are characterized by their small size and minimal cytoplasm in many adult murine and human organs including testis (Figure 3). Absence of LIN and CD45 confirmed that the testicular VSELs are not cells of hematopoietic lineage. Also, these cells stained for phalloidin indicating presence of actin in these cells (Figure 3G and H). Presence of actin indicates that VSELs are not any reminiscent nuclear fragments but intact cells. The smears from busulphan treated testis showed presence of cells less

Primer	Sequence (5'-3')	Annealing
Oct4 F<br>R	AGCTGCTGAAGCAGAAGAGG<br>GGTTCTCATTGTTGTCGGCT	61°C
Oct-4A F<br>R	AACCGTCCCTAGGTGAGCCG<br>CCCACCTGGAGGCCCTTGGAA	63°C
Sca-1 F<br>R	AGAGGAAGTTTTATCTGTGCAGCCC<br>TCCACAATAACTGCTGCCTCCTGA	66°C
Gfra F<br>R	GGCCTACTCGGGACTGATTGG<br>GGGAGGAGCAGCCATTGATTT	58°C
Prohibitin F<br>R	GTGGCGTACAGGACATTGTG<br>AGCTCTCGCTGGGTAATCAA	58°C
Protamine F<br>R	GGCCACCACCACCACAGACACAGGCG<br>TTAGTGATGGTGCCTCCTACATTTCC	66°C
Gapdh F<br>R	GTCCCGTAGACAAAATGGTGA<br>TGCATTGCTGACAATCTTGAG	58°C
Dazl F<br>R	GTGTGTCGAAGGGCTATGGAT<br>ACAGGCAGCTGATATCCAGTG	61°C

Table 1: Primer sequences for various transcripts used in the study.

Citation: Anand S, Bhartiya D, Sriraman K, Patel H, Manjramkar DD (2014) Very Small Embryonic-Like Stem Cells Survive and Restore Spermatogenesis after Busulphan Treatment in Mouse Testis. J Stem Cell Res Ther 4: 216. doi:10.4172/2157-7633.1000216

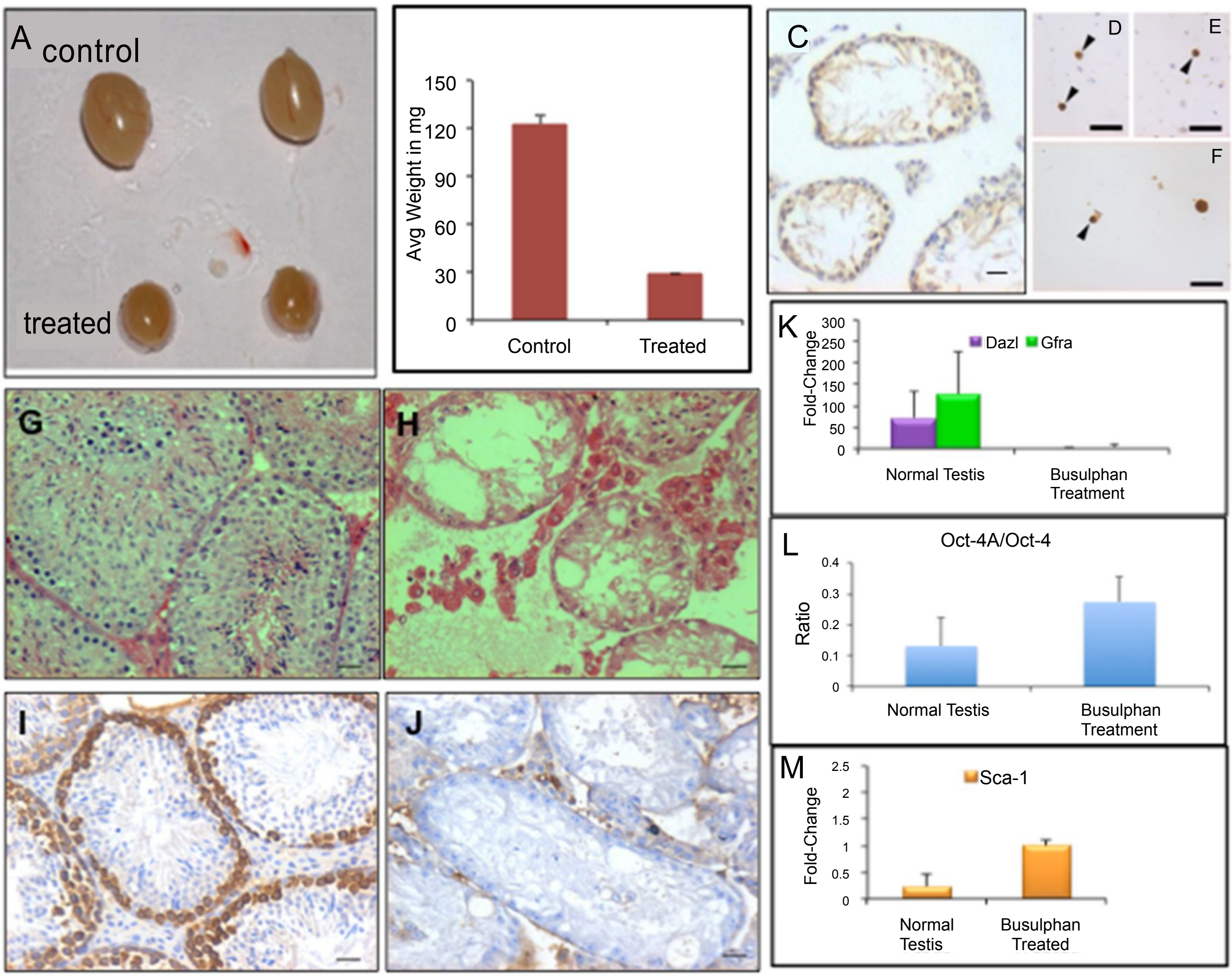

Figure 2: Effect of busulphan treatment on mouse testes: (A) Reduction in size of testes after busulphan treatment. (B) Loss in weight of testes after busulphan treatment.(C) Testicular sections of busulphan treated testis were immuno-stained using OCT-4 antibody to detect the presence of VSELs. Nuclear OCT-4 positive VSELs were detected in germ cells depleted, busulphan treated testicular sections.(D-F) Multiple fields showing nuclear OCT-4 positive VSELs (arrowheads) in busulphan treated testicular smears. H & E stained sections of (G) normal and (H) busulphan treated testes. Note the extensive germ cell depletion and collapsed Sertoli cells after busulphan treatment. Loss of germ cells after busulphan treatment was confirmed by immunolocalization for DAZL. Note the presence of DAZL positive germ cells (spermatogonia and early spermatocytes) in (I)normal testis which are lacking in (J) busulphan treated sections.(K) Differential expression of spermatogonial stem cell (Gfra) and germ cell (Dazl) specific transcripts in testis from control and busulphan treated mice. Note the marked reduction of Dazl ($p<0.01$) and Gfra ($p<0.05$)after busulphan treatment.(L)After busulphan treatment, the proportion of Oct-4A in total Oct-4 as indicated by the ratio of Oct-4A to Oct-4 is increased ($p=0.101$), indicating selective reduction of Oct-4B form. (M) Differential expression of VSELs specific marker Sca-1 transcripts in control and busulphan treated mice testes. Increased Sca-1 expression is observed after busulphan treatment ($p= 0.28$). Error bars represent standard deviation between at least three biological replicates. Bar = 20μm.

than 5um also to be positive for NANOG and SOX-2 (markers for pluripotency, Figure 3I and J). The NANOG positive cells were also positive for STELLA (a primordial germ cell marker, Figure 3K). To the best of our knowledge, this is the first report showing presence of VSELs in chemoablated mice testis.

To further corroborate the immunolocalization results, qRT-PCR analysis was carried out for VSELs specific markers (*Oct-4A* and *Sca-1*) in normal versus busulphan treated testis. *Sca-1*transcripts were increased almost 5 fold after busulphan treatment ($p= 0.280$), suggesting a possible increase in number of VSELs post busulphan treatment (Figure 2M). After busulphan treatment, the proportion of *Oct-4A*in total *Oct-4*as indicated by the ratio of *Oct-4A*to *Oct-4*also increased, although not significantly ($p=0.101$) (Figure 2L). This suggests a selective reduction in cytoplasmic *Oct-4B*form in the total *Oct-4*present after busulphan treatment. These results thus provide further support at mRNA level that the VSELs in testes are spared of the cytotoxic insult due to busulphan treatment and survive.

VSELs were quantitated by flow cytometry in both control and busulphan treated testicular population (Figure 4A and B). Flow cytometry results showed that 0.03% Sca-1^+/LIN$^-$/CD45$^-$cells in normal testis are VSELs while 0.06% VSELs were detected in busulphan treated testis. The percentage of VSELs was significantly higher in busulphan treated animals as compared to control ($p<0.005$) (Figure 4). These cells were sorted and observed under confocal microscope to confirm

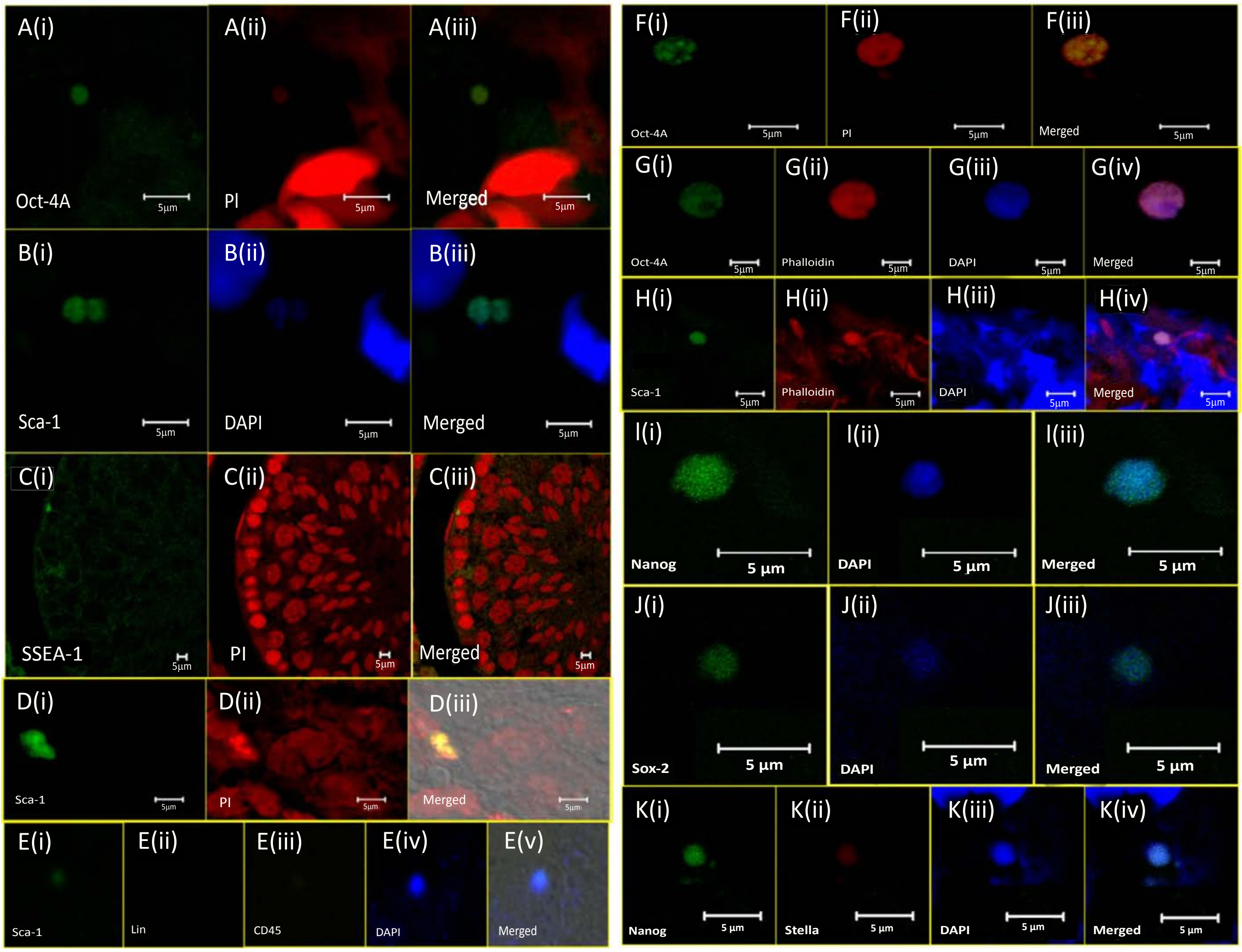

Figure 3: Characterization of testicular VSELs by immunofluorescence: Control mice testicular smears showing presence of nuclear OCT-4A (Ai-iii), SCA-1 (Bi-iii) positive cells. SSEA-1 positive cells were localized next to the basement membrane interspersed between Sertoli cells (Ci-iii) in control mice testicular sections. In busulphan treated mice, localization of SCA-1 positive cells was next to the basement membrane interspersed between Sertoli cells (Di-iii). Note that SCA-1 positive cells were negative for Lin and CD45 (Ei-v). Note also that all the cells observed are in the size range of VSELs i.e. between 2-6um. Busulphan treated mice smears also showed presence of nuclear OCT-4A positive cells (Fi-iii) which also stained positive for phalloidin (Gi-iv). SCA-1 positive cells in busulphan treated testis also stained positive for phalloidin (Hi-iv). Busulphan treated testicular smears also showed presence of NANOG (Ii-iii) and SOX-2 (Ji-iii) positive cells, which are pluripotency markers. NANOG positive cells also stained positive for primordial germ cell marker STELLA (Ki-iv).

the purity of population and the cells were found to express nuclear SCA-1, and were negative for LIN and CD45 (Figure 4C). Mouse bone marrow cells, used as positive control, showed positive staining for both LIN and CD45 (Figure 4D and E). These observations assert that indeed there exists a population of cells expressing pluripotent markers in testis, which are spared of the action of busulphan.

SSCs and VSELs exist as two different populations in mouse testis: In order to further assess whether VSELs are distinct population from spermatogonial stem cells (SSCs), immunostaining and flow cytometry were used. SSCs were immunophenotyped using anti-GFRa antibody. Size of GFRa positive SSCs reported in literature is about 10 um [38] whereas the VSELs are much smaller (2-6 um) in size. Immunophenotyping using GFRa on cells from control testis showed that Gfra positive cells had size greater than 8 µm as observed by shift in PE-positive population and smaller cells were negative for GFRa (Figure 5). In control smears, surface staining for GFRa were observed only in cells larger than the OCT-4A or SCA-1 positive cells (Figure 5A). In smears prepared from busulphan treated animals GFRa staining was not observed. The SCA-1 positive VSELs were found negative for GFRa (Figure 5B and C). Results confirm that VSELs and SSCs are distinct stem cell populations in the mouse testis and that VSELs survive and are spared of busulphan treatment unlike SSCs.

Renewed spermatogenesis from persisting VSELs in chemoablated testis

Effect of transplantation on testicular histology: The testis from vehicle transplanted busulphan treated mice revealed the presence of empty seminiferous tubules (Figure 6) with collapsed Sertoli cells having prominent nucleoli. Sperm were absent in the small, uniform sized seminiferous tubules. There were no signs (minimal at places) of endogenous resumption of spermatogenesis. Leydig cells present in the interstitium were prominent.

In contrast to the vehicle alone transplantation, Sertoli cell

Citation: Anand S, Bhartiya D, Sriraman K, Patel H, Manjramkar DD (2014) Very Small Embryonic-Like Stem Cells Survive and Restore Spermatogenesis after Busulphan Treatment in Mouse Testis. J Stem Cell Res Ther 4: 216. doi:10.4172/2157-7633.1000216

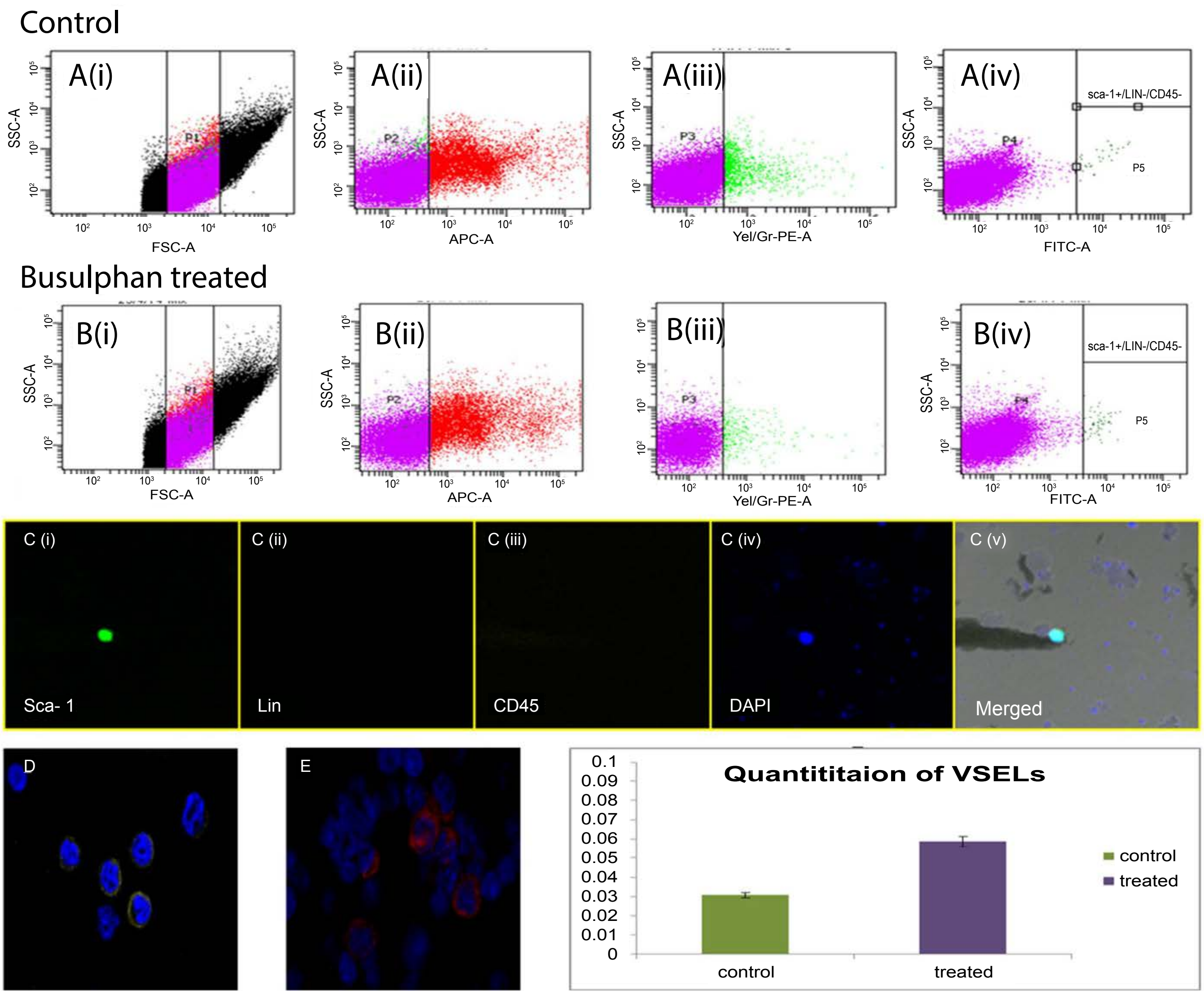

Figure 4: Quantitation of VSELs: Quantitation of VSELs in (A) control and (B) busulphan treated mice testes by flow cytometry. Cells between 2-6 µm were gated using size calibration beads (Ai,Bi) followed by sequential selection of LIN negative population (Aii,Bii), CD45 negative population(Aiii,Biii) and then SCA-1 positive population in both control (Aiv) and busulphan treated (Biv) testes. Confocal images of sorted VSELs show that SCA-1 positive cells were negative for LIN and CD45 (Ci-v). Specificity of APC tagged LIN (D) and PE tagged CD45 (E) antibodies analyzed using mouse bone marrow cell smears as positive control. (F) shows percentage of VSELs obtained in testicular cell suspension prepared from control and busulphan treated mice. Error bars represent standard deviation between three biological replicates.

transplantation revealed the presence of two distinct type of tubules in the testicular sections (Figure 7A and B), bigger tubules with focal spermatogenesis and small sized empty tubules devoid of germ cells. The small, empty tubules comprised of characteristic collapsed Sertoli cells with large cytoplasmic projections into the lumen. These empty tubules were possibly the 'neo-formed tubules' from the transplanted Sertoli cells and resembled the mini-tubules described earlier by Shinohara et al. [39] and Zhang et al. [36]. These mini-tubules located in the close vicinity of the bigger tubules, may serve as a source of various growth factors and cytokines that helped in a paracrine manner to stimulate spermatogenesis from the persisting VSELs in the bigger 'old' native tubules. Surprisingly, a close proximity of Sertoli cells with germ cells did not seem essential for differentiation of VSELs into sperm as evident by absence of Sertoli cells in tubules showing spermatogenesis (Figure 7C, D, F and G). This could have resulted in disorganized nature of spermatogenesis (Figure 7C, F and G) observed in contrast to normal testis (Figure 6A and B). Interestingly, even though Sertoli cells were initially transplanted into one testis alone with the hope that other testis could serve as an ideal vehicle transplantation 'control' group – similar differentiation pattern and appearance of sperm was observed in both the testes. Thus vehicle- transplanted controls were later kept as separate animals.

In the mesenchymal cells transplanted group, similar two populations of tubules viz. the small sized, empty 'neo-formed' tubules and bigger 'native' tubules were observed similar to the Sertoli cell transplanted group (Figure 8A and 8B). However, the Sertoli and Leydig cells appeared more prominent after mesenchymal cells transplantation (Figure 8D and 8E). The 'neo-formed' small sized tubules comprised cells similar to Sertoli cells with prominent nucleoli and abundant cytoplasmic projections into the lumen (Figure 8E).

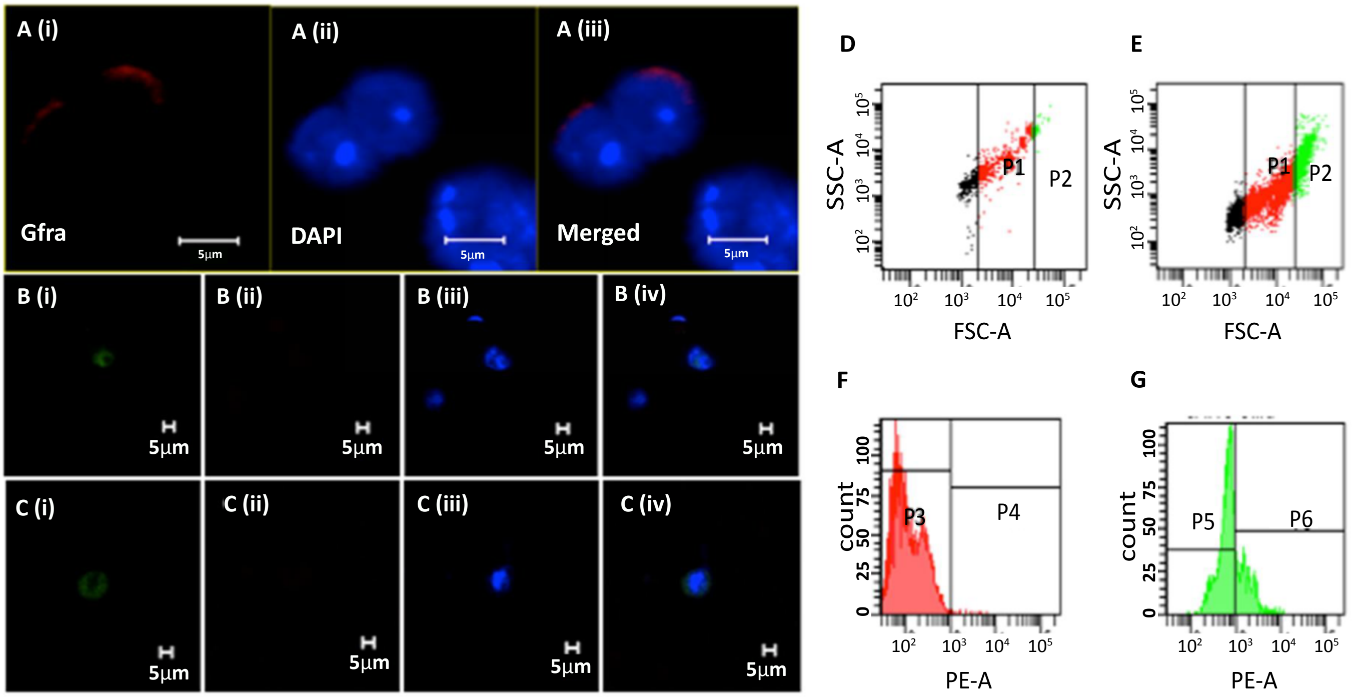

Figure 5: Characterization of mouse testicular stem cells using SCA-1 (for VSELs) and GFRa (for SSCs) (Ai-iii) Cell surface GFRa expression is observed in larger cells in control mice testicular smears. Smaller magnetically separated SCA-1 positive cells were negative for GFRa in both control (Bi-iv) and busulphan treated testicular smears (Ci-iv). (D) Expression of GFRa in larger cells was further validated by flow cytometry based on size calibration beads. P1 includes cells between 2-8 μm and P2 includes cells >8 μm. In control testicular cell suspension (E), GFRa positive cells were seen only in P2 population as observed by the shift in PE positive population i.e. P6 (G) as compared to unstained. No GFRa positive cells are seen in P1 population (F).

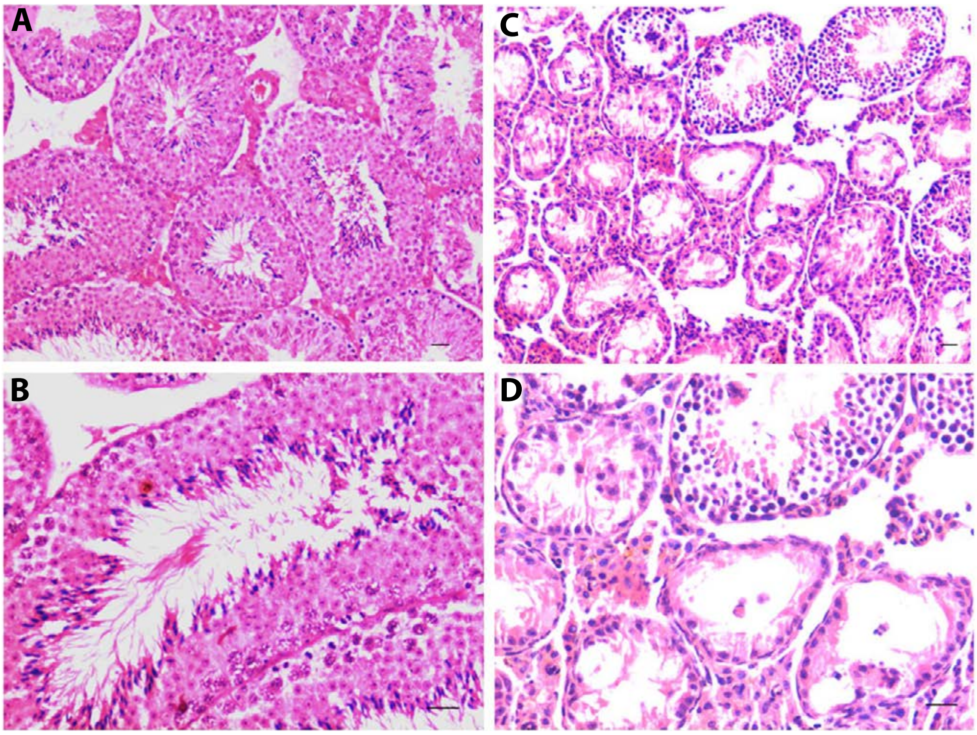

Figure 6: Histological sections of normal and busulphan treated-vehicle transplanted mouse testes. (A,B) Control testicular sections showing normal pattern of spermatogenesis. (C,D) The seminiferous tubules of vehicle transplanted testes, collected at the end of study showed the presence of majority of empty tubules with collapsed Sertoli cells. At places spermatogonial cells were observed in few tubules but sperms were hardly observed. The interstitial compartment comprising Leydig cells was prominent. Bar=20μm.

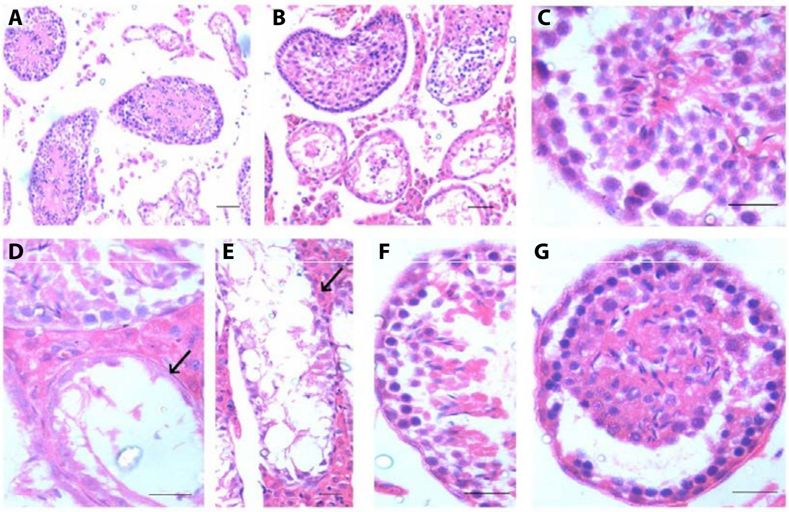

Figure 7: Histological sections of busulphan treated mouse testes after two months of inter-tubular Sertoli cells transplantation. Note the presence of two distinct populations of seminiferous tubules (A,B) including small empty tubules which are possibly the 'neo-formed tubules' from the transplanted Sertoli cells [resemble mini-tubules described earlier by Shinohara et al (38) and Zhang et al (32)] and the bigger 'old' native tubules with focal spermatogenesis and presence of sperm. The sperms are however not arranged in a radial manner (C-G) as in normal testis (Fig 6A,B). The 'neo-formed' tubules are located in close vicinity of bigger tubules (D,E, arrows) and are probably a source of growth factors and cytokines which possibly stimulate the VSELs in the 'old' native tubules to undergo differentiation resulting in sperm production. Interestingly the Sertoli cells are very prominent in the 'neo-formed tubules' and hardly observed in the 'old' tubules. Absence of Sertoli cells in the tubules where spermatogenesis is restored may explain why spermatogenesis is not well organized, but rather remains focal in nature (G). Bar=20µm.

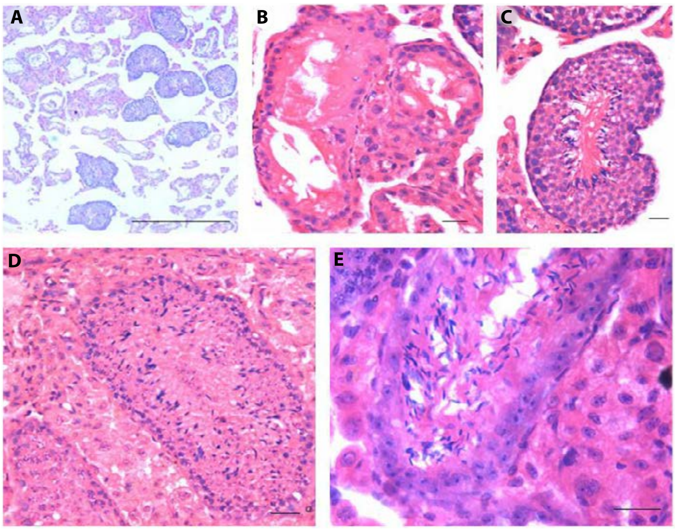

Figure 8: Histological sections of busulphan treated mouse testes after two months of inter-tubular mesenchymal cells transplantation. Similar two kinds of tubules as observed after Sertoli cell transplantation (Figure 7) are observed after transplantation of Mesenchymal cells(A). Interestingly both the interstitial compartment comprising Leydig cells and Sertoli cells in both the small 'neo-formed tubules' and bigger 'native tubules' were much more prominent. Mesenchymal cells apparently differentiated into Sertoli-like cells comprising the 'neo-formed tubules'. The neo-formed and old tubules were observed in close proximity of each other (B). The sperm production was much more and spermatogenesis was much more organized (C-F) compared to after Sertoli cell transplantation (Fig 6 G). This suggests that already 'committed' Sertoli cells have less plasticity compared to Mesenchymal cells to differentiate into tubules which also are a better source of growth factors and cytokines that not only reverts spermatogenesis and sperm production, but also stimulates the somatic compartment comprising Leydig and Sertoli cells. Bar=20µm

The 'old' tubules showed signs of spermatogenesis which appeared more organized (Figure 8C) compared to that observed after Sertoli transplantation.

The number of tubules showing spermatogenesis was significantly higher in the Sertoli cells and Mesenchymal cells transplanted groups in comparison to vehicle transplanted group (Figure 9). This indicated that endogenous restoration of spermatogenesis in vehicle-transplanted animals was not significant (Figure 9A).

Effect of transplantation on testicular germ cells by immuno-localization studies: Two months post transplantation of Sertoli cells or mesenchymal cells, expression levels of stem (PCNA) and germ cell (MVH) markers involved in various stages of spermatogenesis were studied and compared to normal testis and vehicle transplanted group by immuno-localization studies. Normal testicular sections (Figure 10A) showed distinct nuclear localization of PCNA along the basal layer of seminiferous tubules and cytoplasmic expression of MVH in the spermatogonial cells and their descendants (Figure 10G). Post

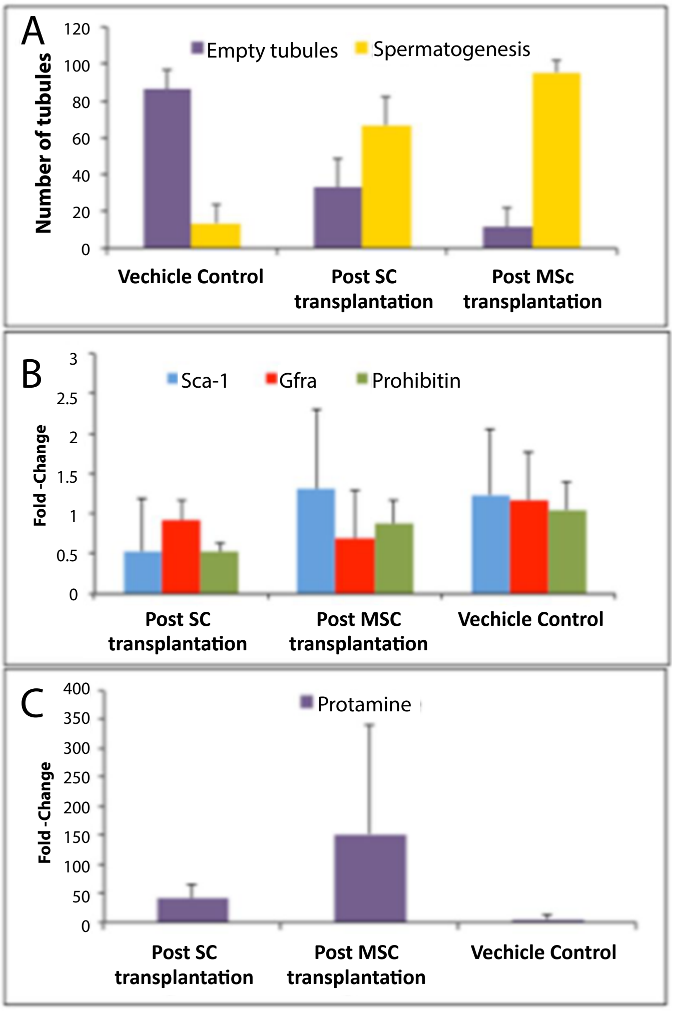

Figure 9: Quantitation of tubules showing spermatogenesis and differential expression of pluripotent and germ cells specific marker transcripts by q-RTPCR, post transplantation of Sertoli cells and Mesenchymal cells compared to vehicle transplanted control. Tubules showing spermatogenesis were counted against the empty tubules and it showed significant increase in number of tubules showing spermatogenesis following Sertoli (SC) ($p<0.005$) and mesenchymal cells (MSC) ($p<0.005$) transplantation (A). The VSELs (Sca-1); spermatogonial (Gfra) and spermatocyte (Prohibitin) specific markers were expressed in all the three groups and no drastic differences were observed ($p<0.05$, $p=0.707$, $p=0.00036$ for Sca-1, Gfra and Prohibitin in Sertoli transplanted group and p= 0.92,0.232, 0.39 for Sca-1, Gfra and Prohibitin in mesenchymal cells transplanted group (B). However, the post-meiotic marker (Protamine) was almost 50 fold up-regulated after Sertoli cells transplantation (p= 0.089) and 150 folds after Mesenchymal cells transplantation ($p<0.05$) compared to vehicle transplanted (C) control. Error bars represent standard deviation between samples and fold change normalized to 18S RNA estimated by qRT-PCR studies in more than 5 animals in each group (two technical replicates for each sample).

transplantation of Sertoli and mesenchymal cells, PCNA positive cells (Figure 10D-10F) and various stages of spermatogenesis (based on the presence of MVH positive spermatogonial cells and their descendants) (Figure 10J-10L) were observed as against vehicle transplanted control sections which were negative for PCNA and MVH (Figure 10B and 10H).Majorly, tubules in vehicle transplanted control sections were negative for MVH staining though few tubules showed MVH staining representing some degree of endogenous restoration (not shown). Figure 10K shows the presence of newly formed tubule devoid of germ cells in the vicinity of a tubule with restored spermatogenesis. It is also intriguing to observe that both number of cells stained positive with PCNA and MVH are much more after mesenchymal cells transplantation compared to Sertoli cells.

Effect of transplantation on testicular gene expression by qRT-PCR studies: Two months post transplantation of Sertoli cells or mesenchymal cells, expression levels of different markers involved in various stages of spermatogenesis were studied and compared to vehicle transplanted group. Levels of Sca-1, Gfra, prohibitin and protamine were analyzed by q-RTPCR (Figure 9B and C). Vehicle treated control showed the presence of VSELs and some degree of pre-meiotic endogenous resumption of spermatogenesis as evident by expression of Sca-1, Gfra and prohibitin transcripts; however protamine, which is transcribed after meiosis in sperm [40] showed minimal expression. The expression levels of Sca-1 and Gfra did not vary significantly ($p<0.05$, $p=0.707$ respectively). However Prohibitin was significantly lower after Sertoli cells transplantation ($p<0.005$) suggesting a transition of spermatocytes to spermatid and later stages which is confirmed by 50 fold higher expression of protamine (post-meiotic marker, p= 0.089). In mesenchymal cells transplanted group, expression levels of Sca-1, Gfra and prohibitin were not drastically altered after transplantation but protamine was up to 150 fold up regulated ($p<0.05$). These results suggest a meiotic block and highlight

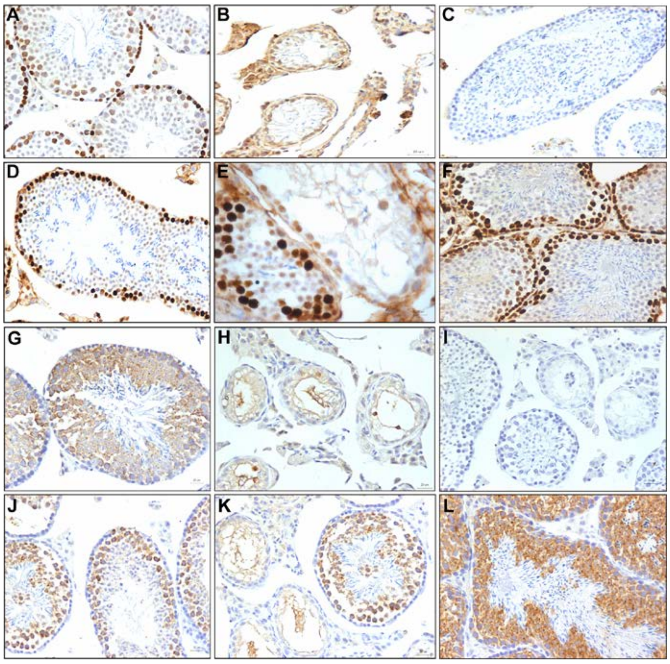

Figure 10: Characterization of spermatogenesis restored after transplantation by immuno-localization studies. Both stem (PCNA, A-F) and germ (MVH, G-L) cell markers were used to study spermatogenesis in chemo-ablated mouse testis after vehicle, Sertoli and mesenchymal cells transplantation. PCNA was expressed in stem cells localized at the basal layer of seminiferous tubules in normal mice (A), Sertoli (D,E) and mesenchymal (F) cells transplantation. Cytoplasmic MVH positive spermatogonial cells and their descendants were detected after Sertoli (J,K) and mesenchymal (L) cells transplantation similar to normal mice (G). Vehicle transplanted control were largely negative for both PCNA (B) and MVH (H). Negative control with omission of primary antibody for PCNA (C) and MVH (I).

the importance of somatic microenvironment in overcoming it and restoring spermatogenesis.

Effect of transplantation on epididymis histology: To evaluate whether the sperm produced in testis progress normally through the epididymis, histology of cauda (a reservoir of sperm prior to ejaculation) was studied. It showed the presence of minimal sperm in vehicle transplanted group (Figure 11 A,B) in contrast to the caudal histological sections that were full of sperm (similar to normal caudal sections, data not shown) in Sertoli (Figure 11C) and Mesenchymal cells transplanted group (Figure 11D).

To further evaluate whether the sperm produced after transplantation of healthy niche cells arise from endogenous cells or from the transplanted cells, Sertoli cells and mesenchymal cells obtained from GFP mice (FVB.Cg-Tg(GFPU) 5NAGY/J) were used for transplantation. The resulting sperm produced after transplantation did not express GFP (Figure 11 I,J) as compared to sperm from GFP mice (Figure 11 H).

Fertilization potential of caudal sperm collected from transplanted group: Our attempts to carry out mating studies using busulphan treated mice could have failed because busulphan exerts a generalized effect on the whole body leading to inability to mate. Hence, *in vitro* fertilization study was undertaken using caudal sperm collected from Sertoli cells or Mesenchymal cells transplanted groups with oocytes obtained from PMSG treated normal mice. Few sperm remained motile even after overnight incubation with the oocytes, few sperm were found to attach to the oocytes and cleavage was observed in both Sertoli and MSC transplanted group (Figure 11 E,F) similar to that seen with sperm of untreated control animals (Figure 11 G). These results suggest that the sperm produced post transplantation possess the ability to fertilize oocytes.

Discussion

Results of the present study demonstrate for the first time that the quiescent very small embryonic-like stem cells (VSELs) present in the mouse testis resist oncotherapy, while the actively dividing progenitors, differentiating germ cells and haploid sperm were depleted due to

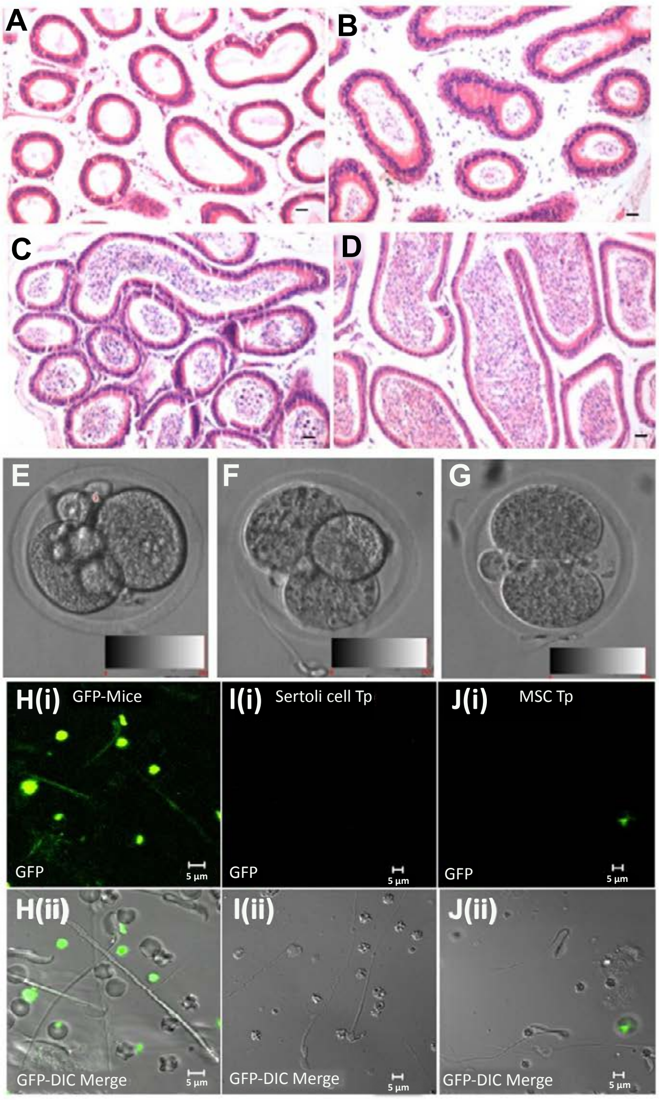

Figure 11: Characterization of spermatogenesis restored after transplantation by immuno-localization studies. Both stem (PCNA, A-F) and germ (MVH, G-L) cell markers were used to study spermatogenesis in chemo-ablated mouse testis after vehicle, Sertoli and mesenchymal cells transplantation. PCNA was expressed in stem cells localized at the basal layer of seminiferous tubules in normal mice (A), Sertoli (D,E) and mesenchymal (F) cells transplantation. Cytoplasmic MVH positive spermatogonial cells and their descendants were detected after Sertoli (J,K) and mesenchymal (L) cells transplantation similar to normal mice (G). Vehicle transplanted control were largely negative for both PCNA (B) and MVH (H). Negative control with omission of primary antibody for PCNA (C) and MVH (I).

busulphan treatment. The VSELs were small in size (2-6 µm), had high nucleo-cytoplasmic ratio, were SCA-1$^+$/CD45$^-$/LIN$^-$and expressed nuclear OCT-4A, SCA-1, NANOG, SOX-2 and STELLA. Quantitation by flow cytometry showed that 0.03% of cells in normal testis and 0.06% cells in busulphan treated testis were VSELs. They comprise a novel population of stem cells (2-6 µm in size and did not express GFRa) distinct from well-studied spermatogonial stem cells (>10 µm in size and expressed GFRa, [38]). Surviving VSELs were not able to differentiate or undergo spermatogenesis in busulphan treated testis possibly because of an altered somatic micro-environment (niche) which is otherwise a source of growth factors and cytokines essential for stem cell differentiation into required lineage [33,34]. Hence attempts were made in the present study to reconstruct the niche by transplanting Sertoli or bone-marrow derived mesenchymal cells by inter-tubular route as against commonly used intra-tubular protocols for testicular transplantation. This approach to restore spermatogenesis in chemoablated testis was successful; resulted in increased population of both testicular stem and germ cells. The sperm produced after transplantation progressed through the epididymis, were detected in large numbers in the cauda and were able to fertilize mouse oocytes *in vitro*. Studies to demonstrate that the sperm produced after transplantation of Sertoli and mesenchymal cells can result in healthy and fertile offspring need to be undertaken. Present study provides new information on testicular stem cells, a novel approach to restore testicular function after chemotherapy and also provides scope for further research which will have relevance in the field of oncofertility.

Our results that VSELs survive chemotherapy in mice testis are in agreement with an earlier report wherein it was shown that mouse bone marrow VSELs persist after total body irradiation, although actively dividing hematopoietic stem cells and differentiated blood cells are completely destroyed [41]. Further studies are warranted to study in details the persisting VSELs after chemotherapy for genetic changes, although it is well known that various oncotherapy regimens mainly target actively dividing cells. It is possible that busulphan treatment affects surviving germ cells and VSELs leading to their inability to undergo spermatogenesis. However, the busulphan treatment used in the study is not lethal suggesting that the busulphan possibly affects only cells where active DNA synthesis occurs [42], which includes the growing tumor and other rapidly dividing testicular cells including SSCs, germ cells and also haploid gametes.

Characteristic features of VSELs observed in the present study are in agreement with the expression profile of VSELs in other tissues reviewed recently [28,43]. Based on marker expression, it has been proposed that primordial germ cells persist into adulthood as VSELs in various body organs [44] including gonads [45]. Presence of VSELs in testes adds another dimension to studies reporting derivation of ES-like colonies from testicular biopsies [32]. However, this is a controversial area of research and limitations exist with respect to efficiency and uniformity of derivation of ES-like colonies [46]. Also, many studies reporting ES-like colonies have used testicular germ cells in culture without enriching SSCs [47,48]. The studies involving initial purification, used markers like Stra8 that are not specific for SSCs [49]. In addition only Conrad's study in humans [50] showed formation of teratoma but the pluripotent colonies reported appears to be a mixture of different cell types and not a pure population [2]. These studies advocate the concept of dedifferentiation of SSCs to pluripotent state; however, certain contradicting studies identify presence of pluripotent population in testis [15,16]. Results of our present study corroborate well with the latter school of thought. In addition to having earlier reported the presence of VSELs in mice [2] and human testes [1] based on marker studies, we have now characterized the testicular VSELs in more details in the present study. VSELs are distinct from the spermatogonial stem cells (SSCs) based on their size and immuno-phenotyping studies. We have also observed that the testicular VSELs are modulated by FSH (personal observations) similar to VSELs in the ovary [4,51,52].

The significance of somatic niche during spermatogenesis has been demonstrated by various studies. Germ cell transplantation studies in mice have shown that spermatogonial stem cells colonize, proliferate and form chains with intercellular bridges but fail to differentiate when transplanted into germ cell ablated mouse testis by intratubular injection through rete testis [53-56]. Brinster's group [33,34] concluded that restoration of fertility in busulphan-treated adult mice is inefficient perhaps because of damage to the testicular micro- environment caused by the ablative therapy. Earlier publications also support this notion since it is well documented that in irradiated rat testes, the surviving stem cells are capable of self-renewal and proliferation, but they are unable to differentiate and undergo apoptosis [57,58]. The significance of the testicular somatic niche comprising of Sertoli cells to support the SSCs activity was realized and hence niche transplantation studies have been attempted in past also.

Shinohara et al. [39] showed that Sertoli cell transplantation leads to their assembly into seminiferous tubule like structures and support spermatogenesis. They further showed that young Sertoli cell niche had a better capacity compared to the adult and that when wild type Sertoli cells were transferred into infertile Steel/Steel^{dickie}mice resulted in spermatogenesis and sperm development. Two years later, Shinohara et al. [59] reported that micro-insemination of Sertoli cells into the testicular efferent duct leads to formation of tubule-like structures and allowed resumption of spermatogenesis and birth of normal offspring through assisted reproductive techniques. Zhang et al. [60] showed that irradiated SSCs could colonize and differentiate in a permissive murine host, but the irradiated rat testes did not support the differentiation of normal, functional, transplanted spermatogonial stem cells. Later on, they reported that a simple interstitial/ intertubular injection of GFP tagged Sertoli cells results in *de novo* tubule-like structures in the testicular interstitium and recovery of endogenous spermatogenesis [36]. It has been earlier suggested that Sertoli cell transplantation along with germ cells may be useful to restore spermatogenesis by providing additional niche to co-transplanted germ cells. Ectopic transplantation of single cell suspension of immature rat testicular cells results in alignment of Sertoli cells as tubules within 2 weeks [61]. Results of present study clearly show that intertubular transplantation of Sertoli cells alone may be sufficient to coax the persisting VSELs to undergo differentiation and sperm production.

The ability of bone marrow derived mesenchymal cells to also reverse differentiation block and restore spermatogenesis, besides the Sertoli cells is indeed stimulating. Mesenchymal cells, also termed mesenchymal stem cells are correctly and alternatively termed as 'multipotent stromal cells' are a rich source of growth factors and cytokines [35]. They are known to be present in all the body organs and tissues [62]. Mesenchymal cells are also considered to be a part of testicular microenvironment [34]. It has been shown that even in the hematopoiesis system; mesenchymal cells from the niche for the hematopoietic stem cells [63]. Results of the present study show that mesenchymal cells derived from bone marrow aid in restoration of spermatogenesis in busulphan treated mice, though the precise mode of action still needs to be determined. The growth factors and cytokines secreted by the mesenchymal cells may have contributed to the observed effect. Alternatively, the anti-inflammatory effect of these cells could

also have brought this effect. However, the dose of busulphan selected by us for depleting germ cells from mice testes results in complete loss of germ cells, thus restoration of spermatogenesis (from persisting germ cells) by suppressing inflammation appears highly unlikely mechanism. Identification of factors secreted by the transplanted cells hence becomes essential.

Based on the results of the present study, we hypothesize that transplantation of autologous bone marrow derived mesenchymal stem cells may benefit the existing azoospermic cancer survivors. This is in agreement with few existing reports on similar concept in the ovaries [64-66]. However whether the bone marrow itself is a source of germ cells is a controversial area in reproductive biology. Johnson et al. [67] reported that bone marrow transplantation could restore oocyte production in busulphan treated adult mice. They also reported that though transplanted bone marrow cells derived oocytes were observed, all the progenies were of recipient origin. This study was contradicted quickly by Eggan et al. [68] who showed that female bone marrow cells failed to produce haploid mature oocytes in parabiotic mouse model. In male mice, ability of bone marrow cells to form germ cells [69] is also controversial. Some reports have shown the transdifferentiation potential of bone marrow cells into germ cells [70-74]. Lue et al. [71] reported that bone marrow stem cells can differentiate into germ cells after transplantation in mouse testis. But Lassalle et al. [74] found no evidence that male bone marrow-derived stem cells undergo spermatogenesis when transplanted into adult mice testis. The difference between the two studies being that Lassalle et al. [74] enriched $SCA1^{+}$side population cells and transplanted intra-tubular into the busulphan treated mouse testis whereas Lue et al. [71] used total adult bone marrow cells and injected into the tubules as well as in the interstitial spaces and obtained differentiation into spermatogonia, spermatocytes as well as Sertoli cells and Leydig cells. This differentiation observed by Lue's group might be because they injected total bone marrow cells thereby having chances of contamination by VSELs, whereas no differentiation observed by Lassalle indicates the compromised niche in recipients that does not support differentiation of VSELs. Lack of differentiation observed by Lasselle suggests the critical requirement for other bone marrow stem cells like mesenchymal cells for restoration of niche and spermatogenesis. Similarly Sabbaghi et al. [73] on transplanting mesenchymal cells intra-tubularly, neither observed expression of cKit (crucial spermatogonial marker essential for spermatogenesis), nor spermatogenesis. Unlike above mentioned studies that favor trans-differentiation, our study and an earlier study [36] clearly show that the recovery of spermatogenesis is from the endogenous cells. In this study, we have demonstrated that the spermatogenesis resumed from endogenous cells and not from germ cell contamination, if any, in transplanted Sertoli cells or trans-differentiation of bone-marrow derived mesenchymal cells through use of cells from GFP mice for transplantation. The sperm were non-GFP and hence must have derived from the persisting VSELs rather than from the transplanted cells. This is in agreement with earlier report by Zhang et al. [36] wherein using GFP tagged Sertoli cells for transplantation, recovery of spermatogenesis was observed only from the endogenous cells. Hence this model of niche transplantation may have significant implications, though more studies will be essential to delineate the precise mechanisms underlying this process.

To conclude, testicular stem cells survive in germ cells depleted mouse testis and can be explored to restore fertility by reconstructing the somatic niche. In a parallel study in mouse ovaries, we have also observed that ovarian VSELs survive in chemoablated ovaries and they show ability to form oocytes (personal observations). Our collaborators have shown (unpublished data) that similar to the results in the present study, VSELs persist in testicular biopsy collected from azoospermic young-adult survivors of childhood cancer having different types of cancer and exposed to different oncotherapy regimens.

Our results provide altogether a new perspective to the field of fertility restoration for cancer survivors and opens up exciting avenues that need to be further explored. The approach used in the present study to restore fertility may have far reaching consequences due to its relatively simple and non-invasive nature compared to technically demanding micro-injections of SSCs into the seminiferous tubules [75,76] and also lesser concerns including 'epigenetic defects' that are normally associated with *in vitro* differentiated 'synthetic' gametes [76]. More studies in this direction from clinical point of view are required in order to harness the possibility of restoring fertility to achieve biological parenthood especially in cancer survivors.

References

1. Bhartiya D, Kasiviswanathan S, Unni SK, Pethe P, Dhabalia JV, et al. (2010) Newer insights into premeiotic development of germ cells in adult human testis using Oct-4 as a stem cell marker. J Histochem Cytochem 58: 1093-1106. [PubMed]

2. Bhartiya D, Kasiviswananthan S, Shaikh A (2012) Cellular origin of testis-derived pluripotent stem cells: a case for very small embryonic-like stem cells. Stem Cells Dev 21: 670-674. [PubMed]

3. Parte S, Bhartiya D, Telang J, Daithankar V, Salvi V, et al. (2011) Detection, characterization, and spontaneous differentiation in vitro of very small embryonic-like putative stem cells in adult mammalian ovary. Stem Cells Dev 20: 1451-1464. [PubMed]

4. Bhartiya D, Sriraman K, Parte S (2012) Stem cell interaction with somatic niche may hold the key to fertility restoration in cancer patients. Obstet Gynecol Int 2012: 921082. [PubMed]

5. Li L, Clevers H (2010) Coexistence of quiescent and active adult stem cells in mammals. Science 327: 542-545. [PubMed]

6. Mascre´ G, Dekoninck S, Drogat B, Youssef KK, Brohee´ S, et al. (2012) Distinct contribution of stem and progenitor cells to epidermal maintenance. Nature 489: 257-262. [PubMed]

7. De Rosa L, De Luca M (2012) Cell biology: dormant and restless skin stem cells. Nature 489: 215-217. [PubMed]

8. Zuccotti M, Merico V, Bellone M, Mulas F, Sacchi L, et al. (2011) Gatekeeper of pluripotency: A common Oct-4 transcriptional network operates in mouse eggs and embryonic stem cells. BMC Genomics 12: 1-13. [PubMed]

9. Cauffman G, Liebaers I, Van Steirteghem A, Van de Velde H (2006) POU5F1 isoforms show different expression patterns in human embryonic stem cells and preimplantation embryos. Stem Cells 24: 2685-2691. [PubMed]

10. Lee J, Kim HK, Rho JY, Han YM, Kim J (2006) The human OCT-4 isoforms differ in their ability to confer self-renewal. J Biol Chem 281: 33554-33565. [PubMed]

11. Liedtke S, Stephan M, Kögler G (2008) Oct4 expression revisited: potential pitfalls for data misinterpretation in stem cell research. Biol Chem 389: 845-850. [PubMed]

12. Wang X, Dai J (2010) Isoforms of OCT4 contribute to the confusing diversity in stem cell biology. Stem Cells 28: 885-893. [PubMed]

13. Ratajczak MZ, Liu R, Ratajczak J, Kucia M, Shin DM (2011) The role of pluripotent embryonic-like stem cells residing in adult tissues in regeneration and longevity. Differentiation 81: 153-161. [PubMed]

14. Ratajczak MZ, Suszynska M, Pedziwiatr D, Mierzejewska K, Greco NJ (2012) Umbilical cord blood-derived very small embryonic like stem cells (VSELs) as a source of pluripotent stem cells for regenerative medicine. Pediatr Endocrinol Rev 9: 639-643. [PubMed]

15. Izadyar F, Wong J, Maki C, Pacchiarotti J, Ramos T, et al. (2011) Identification and characterization of repopulating spermatogonial stem cells from the adult human testis. Hum Reprod 26: 1296-1306. [PubMed]

16. Lim JJ, Sung SY, Kim HJ, Song SH, Hong JY, et al. (2010) Long-term

proliferation and characterization of human spermatogonial stem cells obtained from obstructive and non-obstructive azoospermia under exogenous feeder-free culture conditions. Cell Prolif 43: 405-417. [PubMed]

17. Ratajczak MZ, Kucia M, Ratajczak J, Zuba-Surma EK (2009) A multi-instrumental approach to identify and purify very small embryonic like stem cells (VSELs) from adult tissues. Micron 40: 386-393. [PubMed]

18. Mucksová J, Brillard JP, Hejnar J, Poplstein M, Kalina J, et al. (2009) Identification of various testicular cell populations in pubertal and adult cockerels. Anim Reprod Sci 114: 415-422. [PubMed]

19. Lassalle B, Bastos H, Louis JP, Riou L, Testart J, et al. (2004) ''Side population'' cells in adult mouse testis express Bcrp1 gene and are enriched in spermatogonia and germinal stem cells. Development 131: 479-487. [PubMed]

20. Riou L, Bastos H, Lassalle B, Coureuil M, Testart J, et al. (2005) The telomerase activity of adult mouse testis resides in the spermatogonial alpha6-integrin-positive side population enriched in germinal stem cells. Endocrinology 146: 3926-3932. [PubMed]

21. Kubota H, Avarbock MR, Brinster RL (2003) Spermatogonial stem cells share some, but not all, phenotypic and functional characteristics with other stem cells. Proc Natl Acad Sci USA 100: 6487-6492. [PubMed]

22. Grisanti L, Falciatori I, Grasso M, Dovere L, Fera S, et al. (2009) Identification of spermatogonial stem cell subsets by morphological analysis and prospective isolation. Stem Cells 27: 3043-3052. [PubMed]

23. Bhartiya D, Unni S, Parte S, Anand S (2013) Very small embryonic-like stem cells: Implications in reproductive biology. Biomed Res Int 2013: 682326. [PubMed]

24. Ratajczak MZ, Shin DM, Kucia M (2009) Very small embryonic/epiblast-like stem cells: a missing link to support the germ line hypothesis of cancer development? Am J Pathol 174: 1985-1992. [PubMed]

25. Ratajczak MZ, Shin DM, Liu R, Marlicz W, Tarnowski M, et al. (2010) Epiblast/germ line hypothesis of cancer development revisited: lesson from the presence of Oct-4 + cells in adult tissues. Stem Cell Rev 6: 307-316. [PubMed]

26. Rijlaarsdam MA, van Herk HA, Gillis AJ, Stoop H, Jenster G, et al. (2011) Specific detection of OCT3/4 isoform A/B/B1 expression in solid (germ cell) tumours and cell lines: confirmation of OCT3/4 specificity for germ cell tumours. Br J Cancer 105: 854-863. [PubMed]

27. Shaikh A, Bhartiya D (2012) Pluripotent stem cells in bone marrow and cord blood. Publisher – InTech.

28. Suszynska M, Zuba-Surma EK, Maj M, Mierzejewska K, Ratajczak J, et al. (2014) The proper criteria for identification and sorting of very small embryonic-like stem cells, and some nomenclature issues. Stem Cells Dev 23: 702-713. [PubMed]

29. Binas B, Verfaillie CM (2012) Concise review: bone marrow meets blastocyst: lessons from an unlikely encounter. Stem Cells 31: 620-626. [PubMed]

30. Ko K, Wu G, Araúzo-Bravo MJ, Kim J, Francine J, et al. (2011) Autologous pluripotent stem cells generated from adult mouse testicular biopsy. Stem Cell Rev 8: 435-444. [PubMed]

31. Ko K, Reinhardt P, Tapia N, Schneider RK, Araúzo-Bravo MJ, et al. (2011) Brief report: Evaluating the potential of putative pluripotent cells derived from human testis. Stem Cells 29: 1304-1309. [PubMed]

32. Tapia N, Araúzo-Bravo MJ, Ko K, Schöler HR (2011) Concise review: challenging the pluripotency of human testis-derived ESC-like cells. Stem Cells 29: 1165-1169. [PubMed]

33. Brinster CJ, Ryu BY, Avarbock MR, Karagenc L, Brinster RL, et al. (2003) Restoration of fertility by germ cell transplantation requires effective recipient preparation. Biol Reprod 69: 412-420. [PubMed]

34. Oatley JM, Brinster RL (2012) The germline stem cell niche unit in mammalian testes. Physiol Rev 92: 577-595. [PubMed]

35. Gnecchi M, Zhang Z, Ni A, Dzau VJ (2008) Paracrine mechanisms in adult stem cell signaling and therapy. Circ Res 103: 1204-1219. [PubMed]

36. Zhang Z, Shao S, Shetty G, Meistrich ML (2009) Donor sertoli cells transplanted into irradiated rat testes stimulate partial recovery of endogenous spermatogenesis. Reproduction 137: 497-508. [PubMed]

37. Soleimani M, Nadri S (2009) A protocol for isolation and culture of mesenchymal stem cells from mouse bone marrow. Nat Protoc 4: 102-106. [PubMed]

38. Mays-Hoopes LL, Bolen J, Riggs AD, Singer-Sam J (1995) Preparation of spermatogonia, spermatocytes, and round spermatids for analysis of gene expression using fluorescence-activated cell sorting. Biol Reprod 53: 1003-1011. [PubMed]

39. Shinohara T, Orwig KE, Avarbock MR, Brinster RL (2003) Restoration of spermatogenesis in infertile mice by Sertoli cell transplantation. Biol Reprod 68: 1064-1071. [PubMed]

40. Erickson RP, Kramer JM, Rittenhouse J, Salkeld A (1980) Quantitation of mRNAs during mouse spermatogenesis: protamine-like histone andphosphoglycerate kinase-2 mRNAs increase after meiosis. Proc Natl Acad Sci USA 77: 6086-6090. [PubMed]

41. Ratajczak J, Wysoczynski M, Zuba-Surma E, Wan W, Kucia M, et al. (2011). Adult murine bone marrow-derived very small embryonic-like stem cells differentiate into the hematopoietic lineage after co-culture over OP9 stromal cells. Exp Hematol 39: 225-237. [PubMed]

42. Shiraishi A, Sakumi K, Sekiguchi M (2000) Increased susceptibility to chemotherapeutic alkylating agents of mice deficient in DNA repair methyl transferase. Carcinogenesis 21: 1879-1883.

43. Zuba-Surma EK, Kucia M, Wu W, Klich I, Lillard JW Jr, et al. (2008) Very small embryoniclike stem cells are present in adult murine organs: image stream-based morphological analysis and distribution studies Cytometry A 73A: 1116-1127.

44. Ratajczak MZ, Machalinski B, Wojakowski W, Ratajczak J, Kucia M (2007) A hypothesis for an embryonic origin of pluripotent Oct-4(+) stem cells in adult bone marrow and other tissues. Leukemia 21: 860-867. [PubMed]

45. Massasa E, Costa XS, Taylor HS (2010) Failure of the stem cell niche rather than loss of oocyte stem cells in the aging ovary. Aging (Albany NY) 2: 1-2. [PubMed]

46. Chikhovskaya JV, Jonker MJ, Meissner A, Breit TM, Repping S, et al (2012) Human testis-derived embryonic stem cell-like cells are not pluripotent, but possess potential of mesenchymal progenitors. Hum Reprod 27: 210-221. [PubMed]

47. Kossack N, Meneses J, Shefi S, Nguyen HN, Chavez S, et al. (2009) Isolation and characterization of pluripotent human spermatogonial stem cell-derived cells. Stem Cells 27: 138-149. [PubMed]

48. Golestaneh N, Kokkinaki M, Pant D, Jiang J, DeStefano D, et al. (2009) Pluripotent stem cells derived from adult human testes. Stem Cells Dev 18: 1115-1126. [PubMed]

49. Guan K, Nayernia K, Maier LS, Wagner S, Dressel R, et al. (2006) Pluripotency of spermatogonial stem cells from adult mouse testis. Nature 440: 1199-1203. [PubMed]

50. Conrad S, Renninger M, Hennenlotter J, Wiesner T, Just L, et al. (2008) Generation of pluripotent stem cells from adult human testis. Nature 456: 344-349. [PubMed]

51. Patel H, Bhartiya D, Parte S, Gunjal P, Yedurkar S, et al. (2013) Follicle stimulating hormone modulates ovarian stem cells through alternatively spliced receptor variant FSH-R3. J Ovarian Res 6: 52. [PubMed]

52. Parte S, Bhartiya D, Manjramkar DD, Chauhan A, Joshi A (2013) Stimulation of ovarian stem cells by follicle stimulating hormone and basic fibroblast growth factor during cortical tissue culture. J Ovarian Res 6: 20. [PubMed]

53. Brinster RL, Zimmermann JW (1994) Spermatogenesis following male germ cell transplantation. Proc Natl Acad Sci USA 91: 11298-11302. [PubMed]

54. Wu X, Goodyear SM, Abramowitz LK, Bartolomei MS, Tobias JW, et al. (2012). Fertile offspring derived from mouse spermatogonial stem cells cryopreserved for more than 14 years. Hum Reprod 27: 1249-1259. [PubMed]

55. Russell LD, Griswold MD (2000) Spermatogonial transplantation-an update for the millennium. Mol Cell Endocrinol 161: 117-120. [PubMed]

56. Dobrinski I, Avarbock MR, Brinster RL (1999) Transplantation of germ cells from rabbits and dogs into mouse testes. Biol Reprod 61: 1331-1339. [PubMed]

57. Kangasniemi M, Huhtaniemi I, Meistrich ML (1996) Failure of spermatogenesis to recover despite the presence of spermatogonia in the irradiated LBNF1 rat. Biol Reprod 54: 1200-1208. [PubMed]

58. Meistrich ML, Shetty G (2003) Inhibition of spermatogonial differentiation by testosterone. J Androl 24: 135-148. [PubMed]

59. Kanatsu-Shinohara M, Miki H, Inoue K, Ogonuki N, Toyokuni S, et al. (2005)

Germline niche transplantation restores fertility in infertile mice. Hum Reprod 20: 2376-2382. [PubMed]

60. Zhang Z, Shao S, Meistrich ML (2007) The radiation induced block in spermatogonial differentiation is due to damage to the somatic environment, not the germ cells. J Cell Physiol 211: 149-158. [PubMed]

61. Gassei K, Schlatt S, Ehmcke J (2006) De novo morphogenesis of seminiferous tubules from dissociated immature rat testicular cells in xenografts. J Androl 27: 611-618. [PubMed]

62. da Silva Meirelles L, Chagastelles PC, Nardi NB (2006) Mesenchymal stem cells reside in virtually all post-natal organs and tissues. J Cell Sci 119: 2204-2213. [PubMed]

63. Méndez-Ferrer S, Michurina TV, Ferraro F, Mazloom AR, Macarthur BD, et al. (2010) Mesenchymal and haematopoietic stem cells form a unique bone marrow niche. Nature 466: 829-834. [PubMed]

64. Ghadami M, El-Demerdash E, Zhang D, Salama SA, Binhazim AA, et al. (2012) Bone marrow transplantation restores follicular maturation and steroid hormones production in a mouse model for primary ovarian failure. PLoS One 7: e32462.

65. Fu X, He Y, Xie C, Liu W (2008) Bone marrow mesenchymal stem cell transplantation improves ovarian function and structure in rats with chemotherapy-induced ovarian damage. Cytotherapy 10: 353-363. [PubMed]

66. Lee HJ, Selesniemi K, Niikura Y, Niikura T, Klein R, et al. (2007) Bone marrow transplantation generates immature oocytes and rescues long-term fertility in a preclinical mouse model of chemotherapy induced premature ovarian failure. J Clin Oncol 25: 3198-3204. [PubMed]

67. Johnson J, Bagley J, Skaznik-Wikiel M, Lee HJ, Adams GB, et al. (2005) Oocyte generation in adult mammalian ovaries by putative germ cells derived from bone marrow and peripheral blood. Cell 122: 303-315. [PubMed]

68. Eggan K, Jurga S, Gosden R, Min IM, Wagers AJ (2006) Ovulated oocytes in adult mice derive from non-circulating germ cells. Nature 441: 1109-1114. [PubMed]

69. Nayernia K, Lee JH, Drusenheimer N, Nolte J, Wulf G, et al. (2006) Derivation of male germ cells from bone marrow stem cells. Lab Invest 86: 654-663. [PubMed]

70. Drusenheimer N, Wulf G, Nolte J, Lee JH, Dev A, et al. (2007) Putative human male germ cells from bone marrow stem cells. Soc Reprod Fertil Suppl 63: 69-76. [PubMed]

71. Lue Y, Erkkila K, Liu PY, Ma K, Wang C, et al. (2007) Fate of bone marrow stem cells transplanted into the testis: potential implication for men with testicular failure. Am J Pathol 170: 899-908. [PubMed]

72. Aziz MTA, Mostafa T, Atta H, Asaad S, Fouad HH, et al. (2011) In vitro and in vivo lineage conversion of bone marrow stem cells into germ cells in experimental azoospermia in rat. Stem Cell Studies.

73. Sabbaghi MA, Bahrami AR, Behrooz G, Kalantar SM, Matin MM, et al. (2012) Trial evaluation of bone marrow derived mesenchymal stem cells (MSCs) transplantation impacts in testicular torsion spermatogenesis revision. Middle East Fertil Soc J 17: 243-249.

74. Lassalle B, Mouthon MA, Riou L, Barroca V, Coureuil M, et al. (2008) Bone marrow-derived stem cells do not reconstitute spermatogenesis *in vivo*. Stem Cells 26: 1385-1386. [PubMed]

75. Oatley JM, Brinster RL (2006) Spermatogonial stem cells. Methods Enzymol 419: 259-282. [PubMed]

76. Daley GQ (2007) Gametes from embryonic stem cells: a cup half empty or half full? Science 316: 409-410. [PubMed]

Citation: Anand S, Bhartiya D, Sriraman K, Patel H, Manjramkar DD (2014) Very Small Embryonic-Like Stem Cells Survive and Restore Spermatogenesis after Busulphan Treatment in Mouse Testis. J Stem Cell Res Ther 4: 216. doi:10.4172/2157-7633.1000216

Submit your next manuscript and get advantages of OMICS Group submissions

Unique features:

- User friendly/feasible website-translation of your paper to 50 world's leading languages
- Audio Version of published paper
- Digital articles to share and explore

Special features:

- 350 Open Access Journals
- 30,000 editorial team
- 21 days rapid review process
- Quality and quick editorial, review and publication processing
- Indexing at Pubmed (partial), Scopus, EBSCO, Index Copernicus and Google Scholar etc
- Sharing Option: Social Networking Enabled
- Authors, Reviewers and Editors rewarded with online Scientific Credits
- Better discount for your subsequent articles

Submit your manuscript at: http://www.omicsonline.org/submission
